# Anti-rheumatoid arthritis effects of traditional Chinese medicine Fufang Xiaohuoluo pill on collagen-induced arthritis rats and MH7A cells

**DOI:** 10.3389/fphar.2024.1374485

**Published:** 2024-04-29

**Authors:** Qiong Yin, Qian Huang, Hantao Zhang, Xiaodi Zhang, Chunlan Fan, Hongping Wang

**Affiliations:** ^1^ Scientific Research Institute of Beijing Tongrentang Co., Ltd., Beijing, China; ^2^ Beijing Zhongyan Tongrentang Medicine Research and Development Co., Ltd., Beijing, China

**Keywords:** rheumatoid arthritis, Fufang Xiaohuoluo pill, traditional Chinese medicine, pathogenesis, inflammation, MH7A, NF-κB

## Abstract

**Background:**

Fufang Xiaohuoluo pill (FFXHL) is a commonly used prescription in clinical practice for treating rheumatoid arthritis in China, yet its specific mechanism remains unclear. This study aims to elucidate the pharmacological mechanisms of FFXHL using both *in vivo* and *in vitro* experiments.

**Methods:**

The collagen-induced arthritis (CIA) rat model was established to evaluate FFXHL’s therapeutic impact. Parameters that include paw swelling, arthritis scores, and inflammatory markers were examined to assess the anti-inflammatory and analgesic effects of FFXHL. Human fibroblast-like synoviocytes (MH7A cells) is activated by tumour necrosis factor-alpha (TNF-α) were used to explore the anti-inflammatory mechanism on FFXHL.

**Results:**

Our findings indicate that FFXHL effectively reduced paw swelling, joint pain, arthritis scores, and synovial pannus hyperplasia. It also lowered serum levels of TNF-α, interleukin-1β (IL1β), and interleukin-6 (IL-6). Immunohistochemical analysis revealed decreased expression of nuclear factor-kappa B (NF-κB) p65 in FFXHL-treated CIA rat joints. *In vitro* experiments demonstrated FFXHL’s ability to decrease protein secretion of IL-1β and IL-6, suppress mRNA expression of matrix metalloproteinases (MMP) −3, −9, and −13, reduce reactive oxygen species (ROS) levels, and inhibit NF-κB p65 translocation in TNF-α stimulated MH7A cells. FFXHL also suppressed protein levels of extracellular signal-regulated kinase (ERK), c-Jun Nterminal kinase (JNK), p38 MAP kinase (p38), protein kinase B (Akt), p65, inhibitor of kappa B kinase α/β (IKKα/β), Toll-like receptor 4 (TLR4), and myeloid differentiation primary response 88 (MyD88) induced by TNF-α in MH7A cells.

**Conclusion:**

The findings imply that FFXHL exhibits significant anti-inflammatory and antiarthritic effects in both CIA rat models and TNF-α-induced MH7A cells. The potential mechanism involves the inactivation of TLR4/MyD88, mitogen-activated protein kinases (MAPKs), NF-κB, and Akt pathways by FFXHL.

## 1 Introduction

Rheumatoid arthritis (RA) is a chronic and multi-system inflammatory autoimmune disease with a global prevalence ranging from approximately 0.5%–1% (Guo et al., 2018). It can manifest at any age, with a higher frequency occurring between 30 and 50 years old, and women experience the condition three times more often than men (Gonzalez et al., 2007; Guo et al., 2018). Clinical presentations include symmetric joint involvement, characterized by arthralgia, swelling, redness, and potential limitations in range of motion (Guo et al., 2018). While the exact pathogenesis of RA remains incompletely understood, it is generally acknowledged as a multifactorial disease, with genetic, environmental, gender, and age-related factors contributing to its progression (Gonzalez et al., 2007). Pro-inflammatory mediators, such as tumour necrosis factor-α (TNF-α), interleukin (IL)-1, IL-6 and matrix metalloproteinases (MMPs) play a significant role in promoting inflammatory infiltration, synovial hyperplasia, joint destruction, and various comorbidities associated with RA (Zhai et al., 2017; Guo et al., 2018). Furthermore, these cytokines have been well-established to be linked with the nuclear factor-kappa B (NF-κB) signalling pathway (Wang et al., 2020).

Toll-like receptor 4 (TLR4) has been established as having relevance to inflammatory and immune responses in RA (Bai et al., 2020). In the synovial fibroblasts of RA patients, the binding of exogenous ligands to TLR2 or TLR4 enhances the production of pro-inflammatory cytokines and chemokines, such as IL-6 (Tang et al., 2010), instigating cartilage inflammation and degeneration (Lorenz et al., 2013). Myeloid differentiation primary response 88 (MyD88), a cytosolic adaptor, is recruited by Toll-like receptors (TLRs) (Liu et al., 2014). In the canonical TLRs pathway, the TLR4 homodimer interacts with MyD88, initiating a signalling cascade resulting in the activation of key transcription factors, including NF-κB (Bai et al., 2020). The NF-κB signalling pathway exhibits heightened activation within the synovium of individuals with RA and it plays a pivotal role in governing synovial inflammation, hyperplasia, and joint degradation (Hammaker et al., 2003; Hayden and Ghosh, 2011). Under normal cellular conditions, NF-κB is bound to inhibitors of NF-κB (IκBs) in the cytoplasm. Upon exposure to activating factors such as TNF-α, IL-1β, the inhibitor of kappa B kinase (IKK) complex becomes activated, leading to the phosphorylation, ubiquitination, and degradation of IκBs. Subsequently, with IκB degradation, the NF-κB/Rel dimers translocate into the nucleus, instigating gene transcription and fostering the expression of cytokines, inflammatory mediators, and matrix-degrading enzymes (Roman-Blas and Jimenez, 2006; Zhai et al., 2018). Notably, immune products (TNF-α, IL-17, IL-6, etc.) generated by NF-κB can reciprocally activate the NF-κB signalling pathway. This feedback loop sustains continual activity within the NF-κB signalling pathway, and inhibiting it can disrupt this persistent state (Roman-Blas and Jimenez, 2006; Zhai et al., 2018).

Beyond the NF-κB signalling pathway, TLR activation also triggers another pathway, the mitogen-activated protein kinases (MAPKs) pathway, which plays a crucial role in RA development (Westra and Limburg, 2006). The three kinase families of this pathway - extracellular signal-regulated kinase (ERK), c-Jun N-terminal kinase (JNK), and p38MAP kinase (p38) - are notably upregulated in RA (Zhou et al., 2002). The activated MAPK pathway contributes to joint damage and inflammation by mediating the expression of pro-inflammatory cytokines in macrophages and fibroblast-like synoviocytes (FLSs) (Cuschieri and Maier, 2005). Additionally, various studies have indicated that manipulating these kinases or cytokines could be advantageous in RA treatment (Wang et al., 2019; Meng et al., 2021). Hence, targeting TLR4 and the downstream signalling pathways NF-κB and MAPK may represent a potential therapeutic strategy in RA treatment.

While a complete cure for RA remains elusive at present, available treatments focus on symptom alleviation and slowing the disease’s progression. The primary treatment goals for RA involve minimizing symptoms like pain and swelling, preventing bone deformities, and maintaining daily functionality (Lü et al., 2015). Commonly used medications for treating RA include nonsteroidal anti-inflammatory drugs (NSAIDs), glucocorticoids, disease-modifying anti-rheumatic drugs (DMARDs). However, prolonged use of these medications can lead to significant side effects, including gastrointestinal and liver disorders (Bullock et al., 2019). New biological agents, such as antit-TNF drugs infliximab, etanercept and adalimumab, are rapidly effective in retarding the progression of the joint damage caused by RA. They represent a more “direct, precise, and focused” approach to treatment. However, biological agents also had some side effects, including a heightened risk of infections and neurological disorders (Bullock et al., 2019). Hence, there is a pressing need to identify a novel anti-RA medication that is safe, effective, and cost-efficient. Traditional Chinese medicine (TCM), deeply integrated into China’s healthcare system, boasts a long history of effectively treating rheumatoid arthritis. In recent years, TCM has gained increasing global recognition, with specific Chinese medicine metabolites undergoing extensive research and demonstrating promising therapeutic effects (Zhang et al., 2010). Previous studies have demonstrated that the Xiaohuoluo pill exhibits immunosuppressive, anti-oxidative, anti-inflammatory,, and analgesic effects, making it a potential treatment for RA (Pan et al., 2006). Derived from the original Xiaohuoluo pill and guided by other ancient prescriptions and principles of TCM, FFXHL incorporates four additional botanical drugs (Supplementary Tables S1,S2). FFXHL consists of ten botanical drugs, including *Aconiturn carmichaelii* Debeaux. (Ranunculaceae; Aconiti Radix; Chuan Wu in Chinese), *Aconitum kusnezoffii* Reichb. (Ranunculaceae; Aconiti Kusnezoffii Radix; Cao Wu in Chinese), *Arisaema erubescens* (Wall.) Schott (Araceae; Arisaema Cum Bile; Dan Nan Xing in Chinese), *Angelica sinensis* (Oliv.) Diels (Apiaceae; Angelicae Sinensis; Dang Gui in Chinese), *Ligusticum chuanxiong* Hort (Apiaceae; Chuanxiong rhizome; Chuan Xiong in Chinese), *Pheretima aspergillum* (E.Perrier) (Megascolecidae; Pheretima; D i Long in Chinese), *Paeonia lactiflora* Pall. (Paeoniaceae; Paeoniae Radix Alba; Bai Shao in Chinese), Commiphora myrrha Engl. Myrrha (Burseraceae; Myrrha; Mo Yao in Chinese), *Boswellia carterii* Birdw. (Burseraceae; Olibanum; Ru Xiang in Chinese), *Cyperus rotundus* L. (Cyperaceae; Cyperi Rhizoma; Xiang Fu in Chinese). All the ten botanical drugs are crushed into fine powder according to proportions (Supplementary Table S1), sifted, and mixed evenly, then for every 100 g of powder, 110–130 g of refined honey is added to make honey pills. Despite the clinical use of FFXHL pill for RA treatment, there is an absence of pertinent pharmacological and mechanistic research. As a result, this study will employ both *in vivo* and *in vitro* experiments to investigate the mechanisms underlying FFXHL’s efficacy in treating RA.

## 2 Materials and methods

### 2.1 Reagents and materials

FFXHL pills (SFDA approval number Z11020012) was purchased from Beijing Tongrentang Co.,Ltd.Tongrentang Pharmaceutical Factory. Bovine type II collagen (20022), Incomplete Freund’s Adjuvant (IFA) (7002) were bought from Chondrex, Inc. (United States). Dexamethasone was purchased from Guangdong South China Pharmaceutical Group Co., Ltd. (Guangdong, China). Rheumatoid fibroblast-like synoviocyte transformed with SV40 T antigen (MH7A) was obtained from Jennio Biological Technology (Guangzhou, China). RPMI 1640 culture medium (22400071), trypsin, Pencillin-Streptomycin (P/S), and fetal bovine serum (FBS) were bought from Gibco (United States). Enzyme-linked immunosorbent assay (ELISA) were purchased from Sinouk Bio (Bei Jing, China) and Lianke Bio (Hang Zhou, China). TNF-a protein was obtained from PRPTO TECH (United States). TLR4, Myd88, p65, p-p65, IKKα/β, p-JNK1/2/3, JNK1/2/3, were purchased from Abcam (United Kingdom). p-Erk1/2, Erk1/2, p-p38, p38, β-actin, p-IKKα/β, p-IκBα, IκBα, p-Akt, Akt were purchased from CST (United States).

### 2.2 Qualitative and quantitative analysis of FFXHL

FFXHL were accurately weighed and ultra-sonicated with 1 mL of 80% methanol for 20 min. The extract was taken out and centrifuged at 13,000 rpm (4°C) and centrifuge for 10 min. Further dilute the solution 100 times with 50% methanol, and place it into a clean sample bottle for LCMS/MS analysis. At the same time, prepare the standard curve with a 50% methanol aqueous solution. The UPLC–MS/MS system consisted of an QTRAP 5500 (AB Sciex, United States) and a Exion LC AD (AB Sciex, United States). Chromatographic separation was performed on Waters XSelect HSS T3 column (2.1 × 100 mm, 2.5 µm) at 40 C with a flow rate of 0.3 mL/min. The mobile phase consisted of acetonitrile (A) and 0 .05% (v/v) aqueous formic acid (B) with gradient elution as follows: 85%–70% B at 0–2 min, 70%–40% B at 2–6 min, 40%–0% B at 6–8 min, 0%–0% B at 8–10 min, 0%–85% B at 10–10.1 min, 85%-85% B at 10.1–13 min. The injection volume was 5 μL. The mass spectrometer was operated in the multiple reaction monitoring (MRM) mode with electrospray positive/negative ionization (ESI). The ionspray voltage was set at 5.5 kV/4.5 kV, and the temperature was maintained at 500°C. The curtain gas was set at 30 psi; collision gas was set at 9; the nebulizer and the heater gas were set to 55 psi. Quantitative parameters are listed in Supplementary Table S3.

After dissolving 50 mg of the FFXHL in 1 mL of ddH_2_O water, 4 mL of methanol was added for ultrasonic extraction for 20 min After filtered with 0.22 μm filter, the samples were analysed on I-Class (Waters Corporation, United States) equipped with a ACQUITY UPLC HSS T3 (2.1 × 100 mm, 1.8 μm). The mobile phase consisted of 0.1% formic acid in pure water (A) and acetonitrile (B). A gradient elution program was as follows: 0%–0% B on 0–1.5  min, 0%–100% B on 1.5–70  min, 100%–100% B on 70–72 min. The flow rate and the injection volume were set at 0.25 mL/min and 10 μL respectively. The mass spectrometric data was collected by Q-TOF SYNAPT G2Si system (Waters Corporation, United States) in both positive and negative ion modes. The working parameters of mass spectrometry were as follows: the capillary voltage was 3.0  kV, the desolvation gas flow was 600 L/min, the cone gas flow was 50 L/min, the source temperature was 120°C, and the desolvation temperature was 360°C.

### 2.3 Animals and CIA modelling

A total of 60 female Sprague-Dawley (SD) rats (6 weeks old; 160 ± 20 g) were provided by Vital River Laboratory Animal Technology Co., Ltd. (Bei Jing, China) [certificate no. SCXK (Jing) 2016-0011].

The rats were fed and housed in a standard pathogen-free environment with a constant temperature of 22°C ± 2°C, 40%–70% humidity, 12-h light/dark cycles and free access to food and water. All animal experiments were approved by the Animal Ethics Committee of Beijing Zhongyan Tongrentang Medicine Research and Development Co., Ltd. (YJY-2021-081801). All experiments were conducted in accordance with the International Guidelines of the Animal Care and Use Committee.

Bovine type II collagen solution was emulsified with an equal volume of IFA to create a stable emulsion of 1 mg/mL in an ice-cold water bath. Excluding the normal group (control, *n* = 7), 0.2 mL of the emulsion was injected on the tail, as previously described with minor modification (Song et al., 2015). On day 7, 0.1 mL of the emulsion was administered as a booster to establish CIA models, avoiding the original injection sites. The normal group received the same amount of saline. The dosage of FFXHL for humans is 0.2 g/kg/day. Therefore, the dosage was 1.2 g/kg per day (medium dose), 2.4 g/kg per day (high dose) and 0.6 g/kg per day (low dose) for rats. After excluding rats where the model was not successfully established, the remaining rats were randomly divided into five groups (*n* = 7 per group): model group (CIA rats), FFXHL high-dose group (CIA rats with 2.4 g/kg of FFXHL, FH), FFXHL medium-dose group (CIA rats with 1.2 g/kg of FFXHL, FM), FFXHL low-dose group (CIA rats with 0.6 g/kg of FFXHL, FL), and positive drug group (CIA rats with 0.3 mg/kg of dexamethasone, designated as DXM). From day 16, the rats in FFXHL and DXM groups were orally administered FFXHL and DXM once a day, while the normal and CIA group rats received the same volume of drinking water using the same method. After 20 days of treatment, these rats were fasted for overnight (with free access to water) and then sacrificed on day 36 (Supplementary Figure S1).

### 2.4 Measurement of body weight, paw swelling, and arthritis score

On the day 0, the rats underwent measurements for body weight and hind paws volumes to establish baseline values. The hind paws volumes were determined by plethysmometry, and these measurements were continued until the day before the rats were sacrificed, along with assessments of body weight, hot-plate latent pain response test, and arthritis score.

The baseline for the hot-plate latent pain response test and arthritis score was established on the day before administering the drugs. In the hot-plate latent pain response test, all rats were placed on a 55°C ± 0.5°C hot-plate to observe pain responses, including paw licking and jumping. The reaction time was defined as the interval between the moment the animal was placed on the plate and the time it initiated licking its claws or raising its paws.

For the arthritis score, in accordance with the criteria from previous literature (Zhao et al., 2021), a scoring system with four levels was used: 0 for no swelling or redness, 1 for slight erythema or swelling of the toes, 2 for swelling and redness of the paws, 3 for severe redness and swelling of the ankles, and 4 for serious redness and swelling of the ankle, foot, and digits. The total score for each rat was the combined score of both hind paws, with a maximum value capped at 8.

### 2.5 Cell culture

MH7A cells were maintained in 1640 medium, supplemented with 10% FBS and 1%P/S in a CO_2_ incubator at 37°C.

### 2.6 Histopathological evaluation of the ankle joints

The ankle joints subsequently harvested from the rats were fixed in 4% paraformaldehyde. Samples were decalcified in 10% ethylenediaminetetraacetic acid (EDTA) at room temperature (25°C–30°C) for approximately 4 weeks. After decalcification, the tissues were dehydrated by ethanol gradient, cleared in xylene. Finally, the samples were paraffin-embedded, cut into 4 mm slices by using a microtome and stained with hematoxylin-eosin (H&E) for routine histological evaluations with a light microscope (Nikon Eclipse E100, Japan).

### 2.7 Immunohistochemical analyses of NF-κB p65

The paraffin sections were deparaffinised and subsequently treated with a 3% H_2_O_2_ solution at room temperature in the dark for 25 min. Afterward, the sections underwent three washes in PBS (pH 7.4) and were then incubated with 3% BSA at room temperature for 30 min. Following this, the sections were exposed to the primary anti-NF-κB p65 antibody (diluted at 1:200) overnight at 4°C, and subsequently, they were treated with HRP goat anti-rabbit secondary antibody (Servicebio, Wuhan, China) at room temperature for 50 min. The sections were developed using 3,3 diaminobenzidine (DAB) and counterstained with hematoxylin at room temperature. Positive area quantification was performed by calculating the ratio of the positive area to the total tissue area using Image-Pro 6.0 analysis software.

### 2.8 ELISA assay

The blood samples were gathered from abdominal aorta and the samples were centrifuged at 4°C for 20 min (3,000 rpm). The levels of serum TNF-α, IL-1β, IL-10, and IL-6 were detected by kits from Sinouk Bio (Beijing, China). MH7A cells were seeded in 6-well plates, after treatment, culture medium was collected, perform ELISA detection according to the requirements of the kits (Lianke Bio, Zhe Jiang, China).

### 2.9 Cell-viability assay

The MH7A cells were plated in 96-well plates. FFXHL was dissolved in DMSO (final concentration is 0.5%) and subjected to ultrasonic processing. FFXHL concentrations ranging from 0.05 to 1 mg/mL (0.05, 0.1, 0.2, 0.4, 0.8, and 1 mg/mL) were applied to the cells for 24 h before treatment with or without TNF-α (20 ng/mL) for an additional 24 h. Subsequently, 10 μL of cell counting kit-8 (CCK-8) solution (Applygen, Beijing, China) was directly introduced to each well, and after a 2-h incubation, the microplate was read at OD480 using a plate reader (BioTek, VT, United States).

### 2.10 Detection of reactive oxygen species

MH7A cells were cultured in black 96-well plates and stimulated with FFXHL (0.05, 0.1, 0.2 mg/mL) for 24 h, followed by stimulation with TNF-α (20 ng/mL) for an additional 24 h. The experimental procedures were conducted in accordance with the guidelines of CellROX™ Deep Red (Thermo Fisher Scientific, United States). NucBlue™ Live ReadyProbes™ reagent was added at a concentration of 1 drop/mL (Thermo Fisher Scientific, United States), and the cells were then incubated at 37°C for 30 min. After PBS washing, the results were assessed using Operetta CLS High-Content Screening (Revvity, Shanghai, China).

### 2.11 Immunofluorescence assay

MH7A cells cultured in black 96-well plates and subjected to the aforementioned treatment. After treatment, cells were fixed with 4% paraformaldehyde for 15 min at room temperature, permeabilized with 0.1% Triton-X100 for 15 min, and then blocked with 10% goat serum for 1 h at room temperature. Subsequently, the cells were incubated overnight with anti- NF-κB p65 antibody in 1:200 (Abcam, United Kingdom) and then incubated with Alexa Fluor^®^ 488 Goat Anti-Rabbit IgG secondary antibodies (Abcam, United Kingdom) at room temperature for 1 h. After washing in PBS, nuclei were stained by NucBlue™ Live ReadyProbes™ reagent at 1 drop/mL for 15 min. The stained cells were analysed with by Operetta CLS High-Content Screening.

### 2.12 Reverse transcription-quantitative PCR (RT-qPCR)

MH7A cells were plated in 6-well dishes and subjected to the aforementioned treatment. Total RNA extraction was carried out using trizol, followed by reverse transcription of RNA into cDNA using the Revert Aid First Strand cDNA Synthesis Kit (Thermo Fisher Scientific, United States). qPCR reactions were conducted with Fast Start Universal SYBR Green Master (Roche, Switzerland). All reactions were performed using the SLAN-96P fluorescent quantitative PCR instrument (Hongshitech, Shanghai, China). Quantification of results was done using the 2^−ΔΔCT^ method, with GAPDH mRNA serving as the internal control. The primer details are provided in Supplementary Table S4.

### 2.13 Western blot

MH7A cells were seeded in 10 cm culture dishes (430167, Corning, United States) and subjected to the aforementioned treatment. All samples were lysed in RIPA cell lysis buffer (Beyotime, Shanghai, China), containing both phosphatase and protease inhibitors (Roche, Switzerland). Protein concentrations were determined using the BCA Protein Assay Kit (Beyotime, Shanghai, China). Equivalent amounts (70 ug) of each sample was separated by electrophoresis on a 4%–20% SDS-PAGE gel and transferred to PVDF membranes. After blocked with 5% bovine serum albumin for 1 h at room temperature, the membranes were incubation in primary antibody (1:300-1:1000, specific dosage in Supplementary Table S5) overnight at 4°C. The secondary HRP-linked antibody was diluted in 1:5000 (CST) or IRDye secondary antibody was diluted in 1:10000 (LI-COR, United States) and membranes were incubated at room temperature for 1 h. HRP-linked antibody binding was visualized by enhanced chemiluminescence (ECL Prime; Cytiva, China). Blots were imaged using the Odyssey^®^ XF (LICOR, United States) and analysed with ImageJ.

### 2.14 Statistical analysis

All data were presented as mean ± standard deviation (mean ± SEM). Statistical analysis was carried out with SPSS 27.0 and prism 9.0. One-way analysis of variance (ANOVA) with Bonferroni *post hoc* test. The Wilcoxon rank-sum test is used for arthritis score and histopathologic score. *p* < 0.05 was considered as statistically significant difference.

## 3 Results

### 3.1 Qualitative and quantitative analysis of FFXHL

**FIGURE 1:**
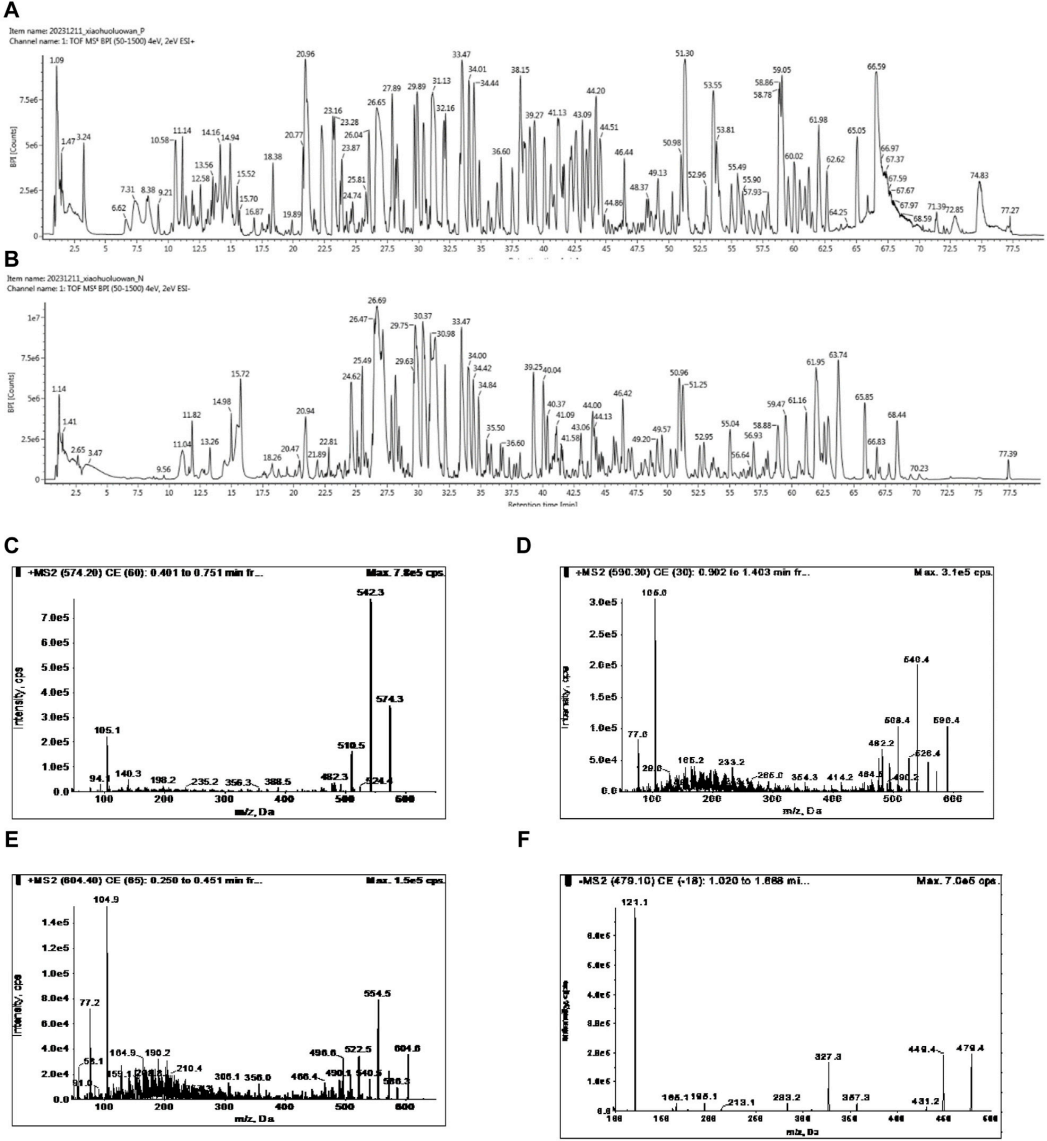
UPLC-MS/MS analysis of chemical compounds in FFXHL in **(A)** Total ion current (TIC) chromatogram of FFXHL under positive ion mode. **(B)** TIC chromatogram of FFXHL under negative ion mode. Tandem mass spectrometry (MS/MS) spectra of **(C)** Benzoylhypacoitine. **(D)** Benzoylmesaconine. **(E)** Benzoylaconitine. **(F)** Paeoniflorin.

The identification and quantification of chemicals are essential for TCM studies to ensure repeatable and consistent results. According to the Chinese Pharmacopoeia - Traditional Chinese Medicine Formulations, benzoylhypacoitine, benzoylmesaconine, benzoylaconitine and paeoniflorin were representative metabolites for quality control of FFXHL. The UPLC-MS/MS was used for the qualitative and quantitative analysis of metabolites in FFXHL. The results of qualitative analysis of FFXHL were shown in Figures 1A, B and Supplementary Table S6. There were total 161 metabolites in FFXHL, including terpenoid, lactone, alkaloid, volatile oils, flavone and amino acid. The contents of benzoylhypacoitine, benzoylmesaconine, benzoylaconitine and paeoniflorin were 0.135 ± 0.002, 0.411 ± 0.004, 0.0781 ± 0.001 and 1.418 ± 0.01 mg/pill, respectively in FFXHL (3 g per pill) (Figures 1D–F).

### 3.2 FFXHL significantly attenuated CIA severity in rats

**FIGURE 2:**
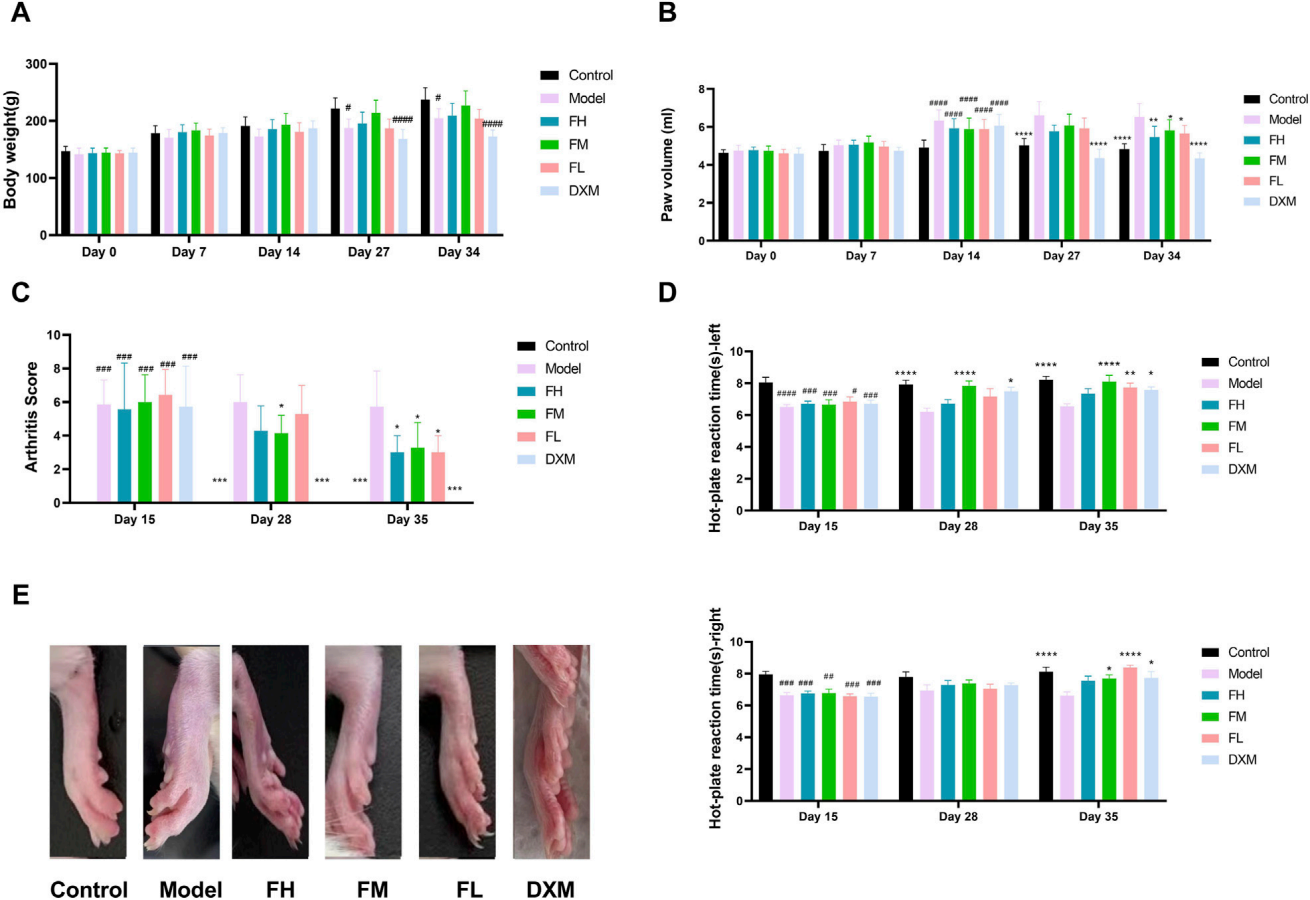
Behavioural assessment of anti-arthritic activity of FFXHL in the CIA rats. **(A)** Body weight. **(B)** Paw swelling. **(C)** Arthritis Score. Hind paws assigned a severity score of 0–4. **(D)** Hot-plate reaction times right and left. **(E)** Macroscopic evidences of arthritis in different groups of rats were observed on day 36. The data are expressed as means ± SEM, ^#^ *p* < 0.05, ^##^ *p* < 0.01, ^###^ *p* < 0.001, ^####^ *p* < 0.0001 vs. control group; *p < 0.05, **p < 0.01, ***p < 0.001, ****p < 0.0001 vs. Model, *n* = 7. FH: FFXHL high dose; FM: FFXHL medium dose; FL: FFXHL low dose.

To investigate the therapeutic effect of FFXHL on RA, a CIA model was induced in rats. Throughout the entire experiment, paw swelling, body weights, and arthritic indexes were assessed. In comparison to the control group, a significant decrease in body weight was observed in the model group and DXM group at days 27 and 34 (Figure 2A). However, rats treated with FFXHL did not show a significant weight increase compared to the CIA model rats throughout the test, with an increasing trend observed in the FM group, though not statistically significant (Figure 2A).

After two injections, all groups, except the control group, displayed significant swelling of the feet (Figures 2B,E). Following treatment with different concentrations of FFXHL, the paw swelling in FH, FM, and FL groups markedly diminished by the final day (Figures 2B,E). In comparison to the FFXHL treatment groups, the DXM treatment group exhibited a more favourable outcome, as DXM is the first-line medication for RA, and its therapeutic effect was evident on day 27, earlier than FFXHL (Figures 2B,E). Consistent with the paw swelling results, arthritis scores also demonstrated a decreasing trend after rats received FFXHL treatment (Figure 2C).

Joint pain is another symptom of RA, and therefore, the pain-relieving effect of FFXHL during RA occurrence was tested. Figure 2D showed that, compared to the model groups, the latency time in FM and FL groups was significantly prolonged in both hind paws. Although the FH group increased the latency time, the results were not significant.

### 3.3 FFXHL improved the histological changes on CIA rats

**FIGURE 3:**
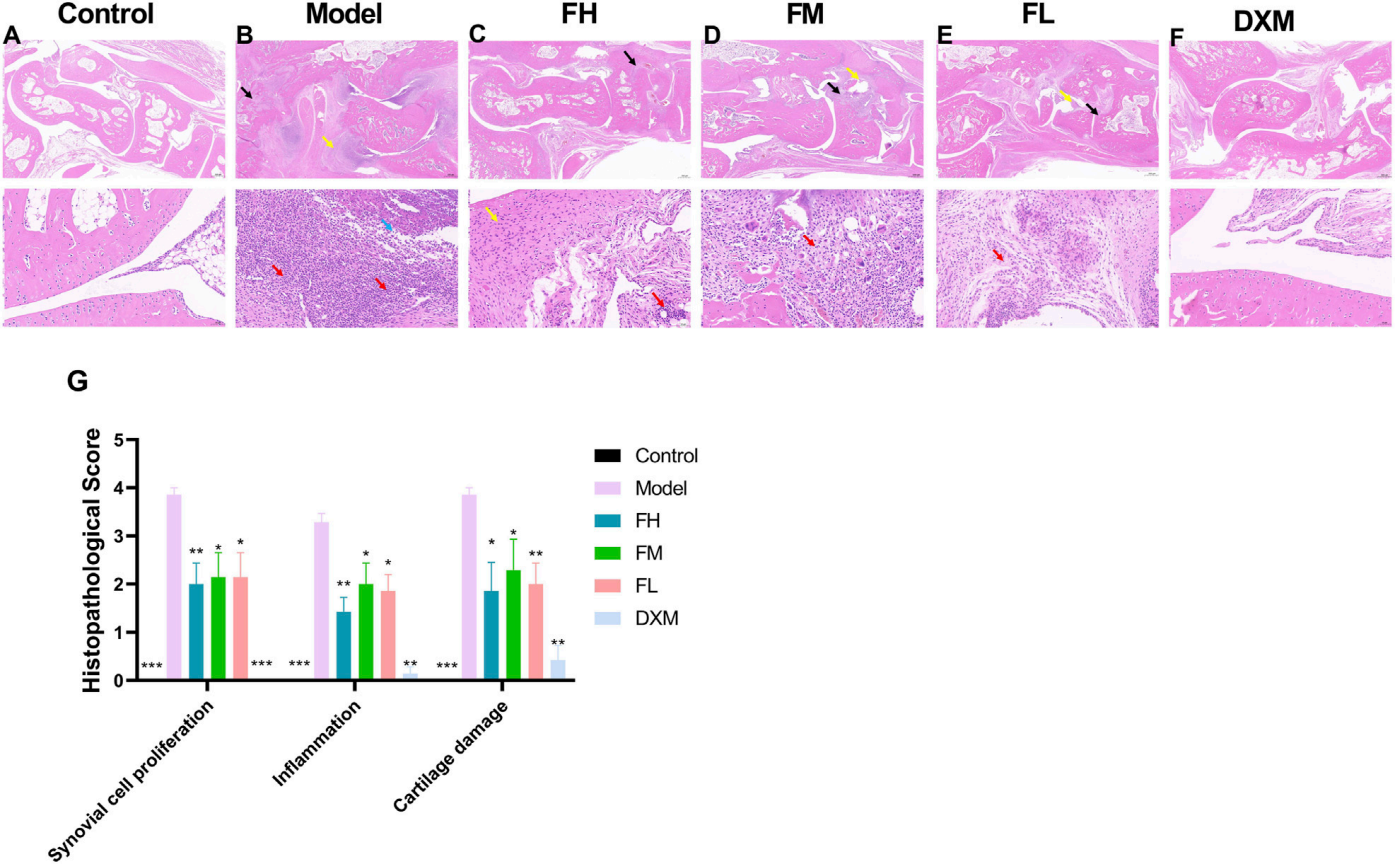
The effect of FFXHL on histological changes of the ankle joints. **(A–F)** Representative pathological sections of ankle joints stained with H & E in different groups (2.0x up and 20.0x down). Control group shows clear and smooth tissues; model groups exhibited severe synovial cell proliferation, inflammatory cellular infiltration, and cartilage damage; FFXHL groups inhibited synovial membrane hyperplasia and reduced the infiltration of inflammatory cells and cartilage injury compared to the model group. **(G)** Pathological alterations were evaluated based on synovial cell proliferation, inflammation and cartilage damage by using semi-quantitative histology scoring. The data are expressed as means ± SEM. *p < 0.05, **p < 0.01, ***p < 0.001 vs. Model, *n* = 7. FH: FFXHL high dose; FM: FFXHL medium dose; FL: FFXHL low dose.

To evaluate the impact of FFXHL on joint damage in CIA rats, histopathological changes were examined, including synovial cell proliferation, inflammation, and cartilage damage. As shown in Figure 3A, the articular cartilage surface in the control group was smooth, the morphology and structure of the chondrocytes are normal, without noticeable synovial hyperplasia or inflammation in the synovial tissue. In contrast, the model groups (Figure 3B) exhibited large amount of bone tissue necrosis and dissolution visible, replaced by extensively proliferated connective tissue (black arrow). There is severe proliferation of the synovium on both sides (yellow arrow) and severe inflammatory cells infiltration (red arrows) and some of them have infiltrated into the joint cavity (blue arrow). Treatment with FFXHL (Figures 3C,D,E) and DXM (Figure 3F)markedly reduced these histological abnormalities (Figure 3G). In FH, FM and FL groups, there was a noticeable reduction in bone necrosis and dissolution. Additionally, these groups showed minimal synovial thickening and only a modest presence of infiltrative inflammatory cells.

### 3.4 The effects of FFXHL on pro and anti-inflammatory cytokines

**FIGURE 4:**
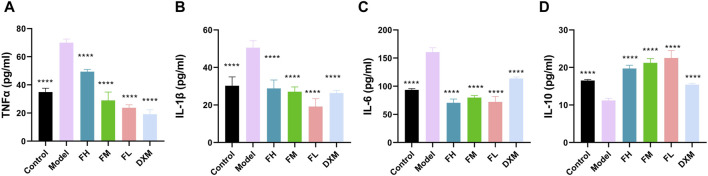
Concentrations of **(A)** TNF-α, **(B)** IL-1 β **(C)** IL-6, and **(D)** IL-10 in serum of each group were determined via ELISA. The data are expressed as means ± SEM. ****p < 0.0001 vs. Model. *n* = 7. FH: FFXHL high dose; FM: FFXHL medium dose; FL: FFXHL low dose.

The expression levels of TNF-α, IL-1β, IL-6, and IL-10 in serum from different groups were subsequently examined. Compared with the control group, the serum levels of TNF-α, IL-1β, and IL-6 were significantly increased in the model group (Figures 4A–C). All FFXHL-treated rats exhibited effectively reduced levels of TNF-α, IL-1β, and IL-6, indicating an anti-inflammatory role of FFXLH in CIA model rats. IL-10, as an anti-inflammatory cytokine, showed a sharp reduction trend in model groups; however, after FFXHL treatment, the serum level of IL-10 increased significantly (Figure 4D).

### 3.5 The anti-inflammatory effects of FFXHL was mediated by inhibition of NF-κB

**FIGURE 5:**
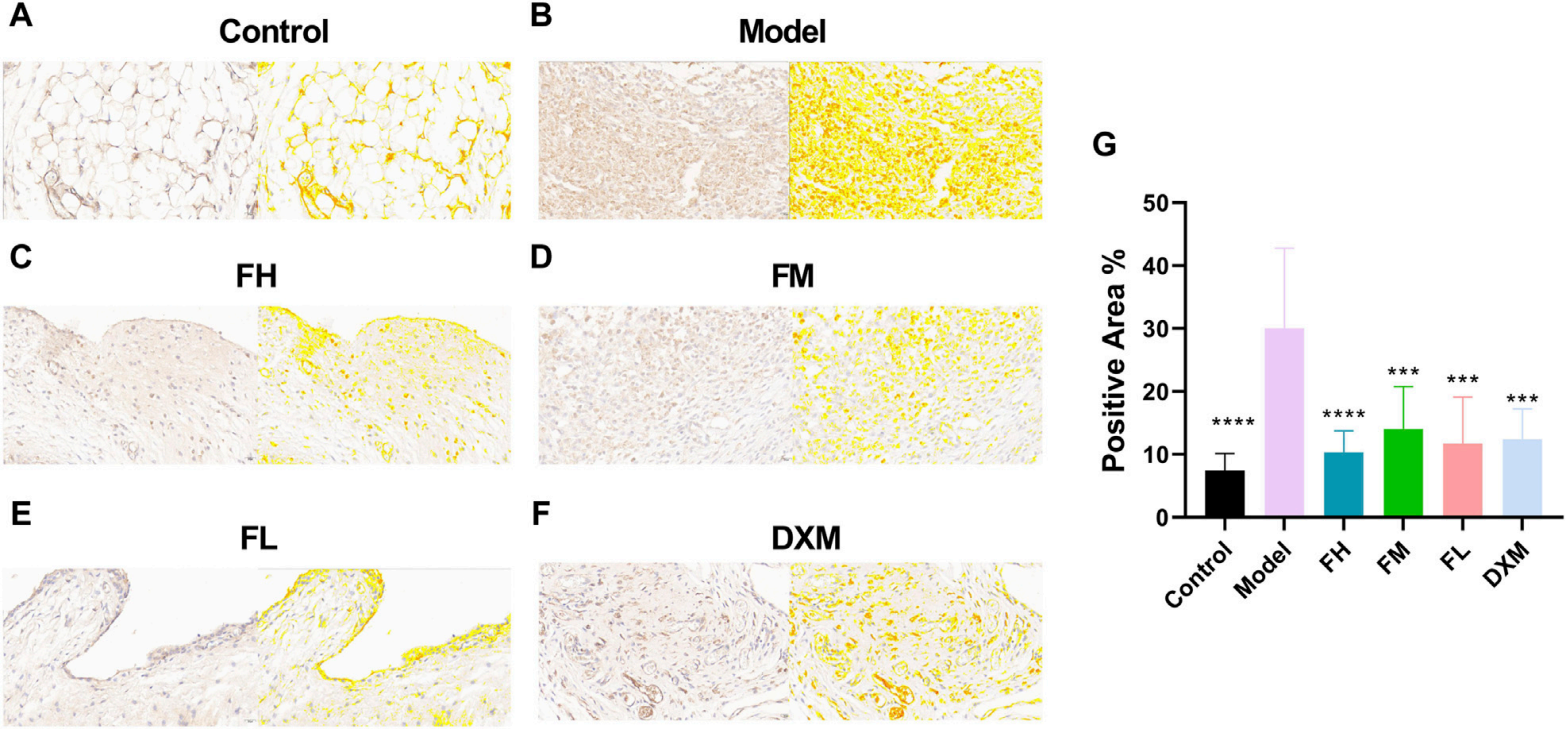
Immunohistochemistry analysis of NF-κB p65 in the joint of CIA rats. The pictures **(A–F)** Displayed immunohistochemical sections of the joints from the different treatment groups for NF-κB p65 (40.0x). Immunohistochemistry analysis was quantified by measuring the mean optical density (OD) of each image. **(G)** Immunohistochemistry analysis were quantified by measuring the positive area of each image. Values were presented as means ± SEM. **p < 0.01, ***p < 0.001, ****p < 0.0001 vs. Model. *n* = 6. FH: FFXHL high dose; FM: FFXHL medium dose; FL: FFXHL low dose.

The effects of FFXHL on NF-κB p65 expression in the joints of CIA rats was immunohistochemically examined. As shown in Figures 5A,B, NF-κB p65 was strongly expressed in synovium of the CIA rats, while in control rats, there were barely positive signals can be observed. After treatment with FFXHL and DXM, the positive signals of NF-κB p65 in the synovium of CIA rats were limited and weak (Figures 5C–G).

### 3.6 FFXHL inhibited stimulated MH7A cells proliferation

**FIGURE 6:**
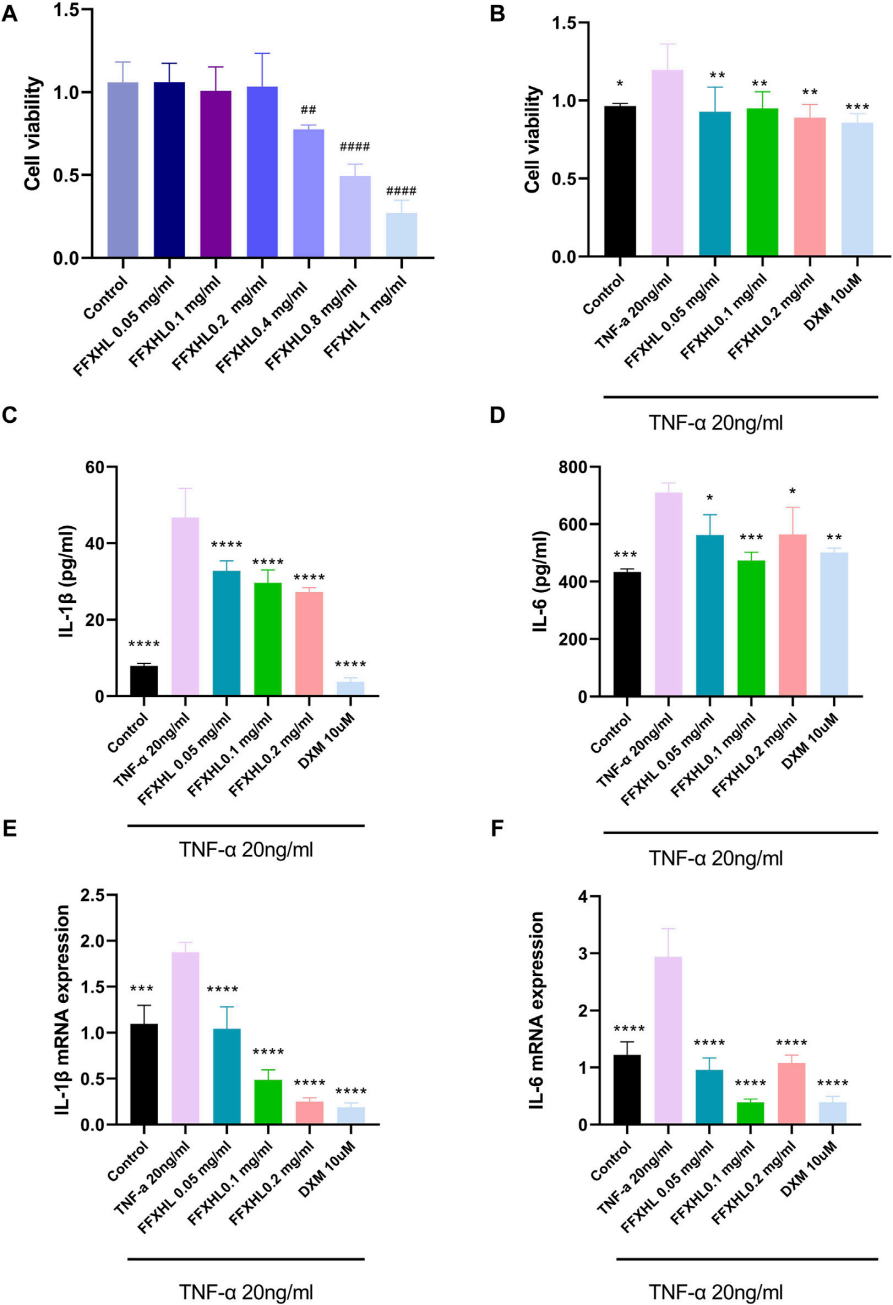
Effects of FFXHL on TNF-α induced pro-inflammatory cytokine production and cell proliferation. For A, MH7A cells treated with different concentrations of FFXHL for 24 h. For B–G, MH7A cells were pre-treated with FFXHL for 24 h and then stimulated with 20 ng/mL TNF-α. **(A)** The effect of different concentrations FFXHL on MH7A cell viability. **(B)** The effects of FFXHL on TNF-α induced cell proliferation. **(C–D)** Effects of FFXHL on TNF-α induced pro-inflammatory cytokine IL-1β and IL-6 production. **(E–F)** The mRNA expression of IL-1β and IL-6. The data are expressed as means ± SEM, ^##^ *p* < 0.01, ^####^ *p* < 0.0001 vs. control; *p < 0.05, **p < 0.01, ***p < 0.001, ****p < 0.0001 vs. TNF-α 20 ng/mL.

To further understand the possible underlying mechanism of FFXHL on treatment RA, an *in vitro* models of cell was employed. The MH7A synovial fibroblast cell line is derived from the synovium of a female patient with RA and has been chemically modified using the SV40 T antigen. This cell line is capable of stable propagation across 10 to 15 passages. Its use has significantly facilitated the screening of drugs for RA and the investigation into the mechanisms of action of RA treatments in recent years (Dai et al., 2022). Firstly, the potential cytotoxicity of FFXHL on MH7A cells was evaluated. Different concentrations (0.05, 0.1, 0.2, 0.4, 0.8, and 1 mg/mL) of FFXHL were added to MH7A cells for 24 h, and cell viability was tested using CCK8 (Figure 6A). The results demonstrated that treatment with FFXHL at concentrations of 0.05, 0.1, and 0.2 mg/mL had no effect on cell viability, so these three concentrations would be used in the next step. Next, to investigate the effects of FFXHL on TNF-α-stimulated cell proliferation, 0.05, 0.1, and 0.2 mg/mL FFXHL were administered to cells for 24 h before 20 ng/mL TNF-α stimulation. TNF-α is a major inflammatory mediator in the pathogenesis of RA as it can promote RA symptoms like secretion of inflammatory cytokines and fibroblast proliferation. Therefore, TNF-α is frequently utilized *in vitro* to treat fibroblasts in order to stimulate RA (Dai et al., 2022). The results showed that after 24 h of treatment with TNF-α, MH7A cells proliferated significantly, and FFXHL significantly inhibited this cell proliferation (Figure 6B).

### 3.7 FFXHL suppresses the expression of pro-inflammatory factors in TNF-α stimulated MH7A cells

In order to create an *in vitro* system for evaluating anti-inflammatory activity, MH7A cells were stimulated with 20 ng/mL TNF-α. After 24 h, there was a significant increase in the expression of IL-1 and IL-6 in the culture supernatant. However, 24 h of FFXHL treatment reduced the release of IL-1β and IL-6 in the supernatant, as well as the mRNA expression in TNF-α-stimulated MH7A cells (Figures 6C–F).

### 3.8 FFXHL suppresses the ROS level in TNF-α stimulated MH7A cells

**FIGURE 7:**
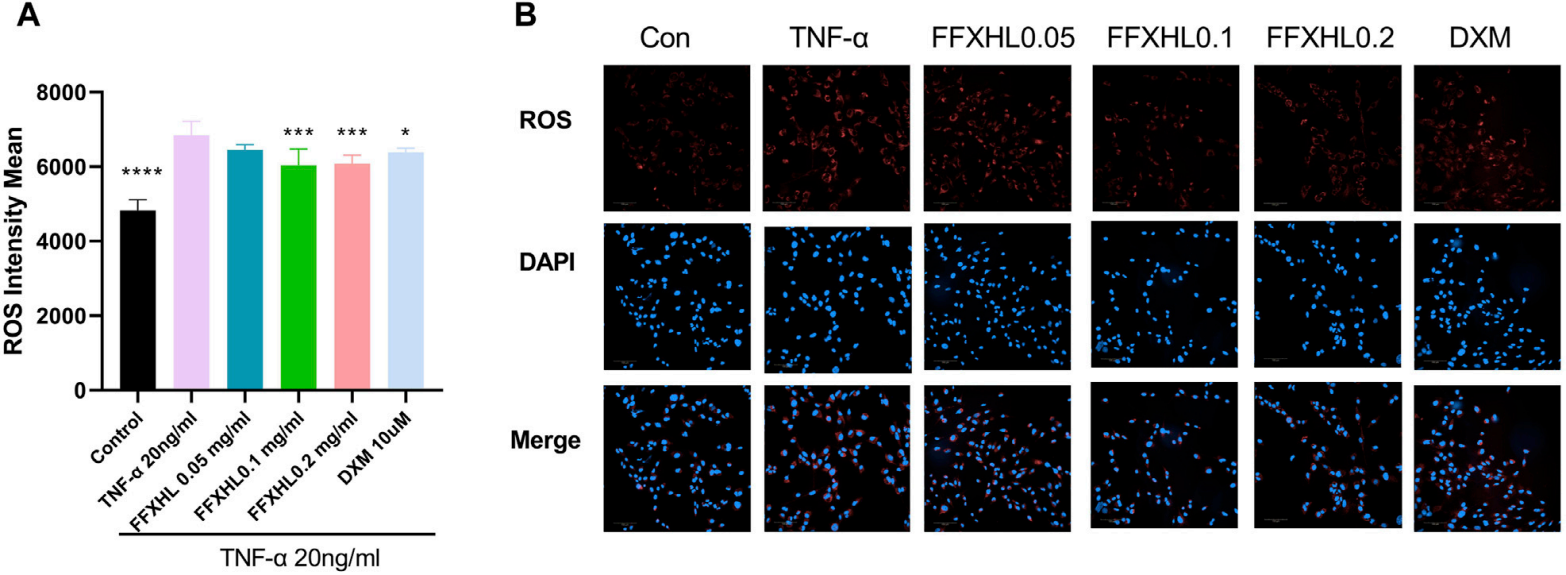
FFXHL decreased ROS level in TNF-α induced cells. MH7A cells were pre-treated with different concentrations of FFXHL for 24 h, then the cells were stimulated with 20 ng/mL TNF-α for 24 h. Detection of ROS levels in MH7A cells in the four groups was analysed by Operetta CLS High Content Screening. Scale bar, 100 μm. The data are expressed as means ± SEM, ****p < 0.0001 vs. TNF-α 20 ng/mL.

Reactive oxygen species (ROS) contribute to the destruction of collagen tissues, and a high level of ROS is associated with damage leading to cartilage degradation (Ahsan et al., 2022). To further elucidate the role of ROS in TNF-α-stimulated inflammation of MH7A cells, cells were pre-treated with FFXHL (0.05, 0.1, 0.2 mg/mL) for 24 h and then stimulated with 20 ng/mL TNF-α. The results showed that TNF-α significantly increased the intracellular ROS level (Figures 7A,B); 0.1 and 0.2 mg/mL FFXHL effectively reduced the accumulation of ROS in MH7A cells.

### 3.9 FFXHL inhibits NF-κB p65 nuclear translocation in TNF-α stimulated MH7A cells

**FIGURE 8:**
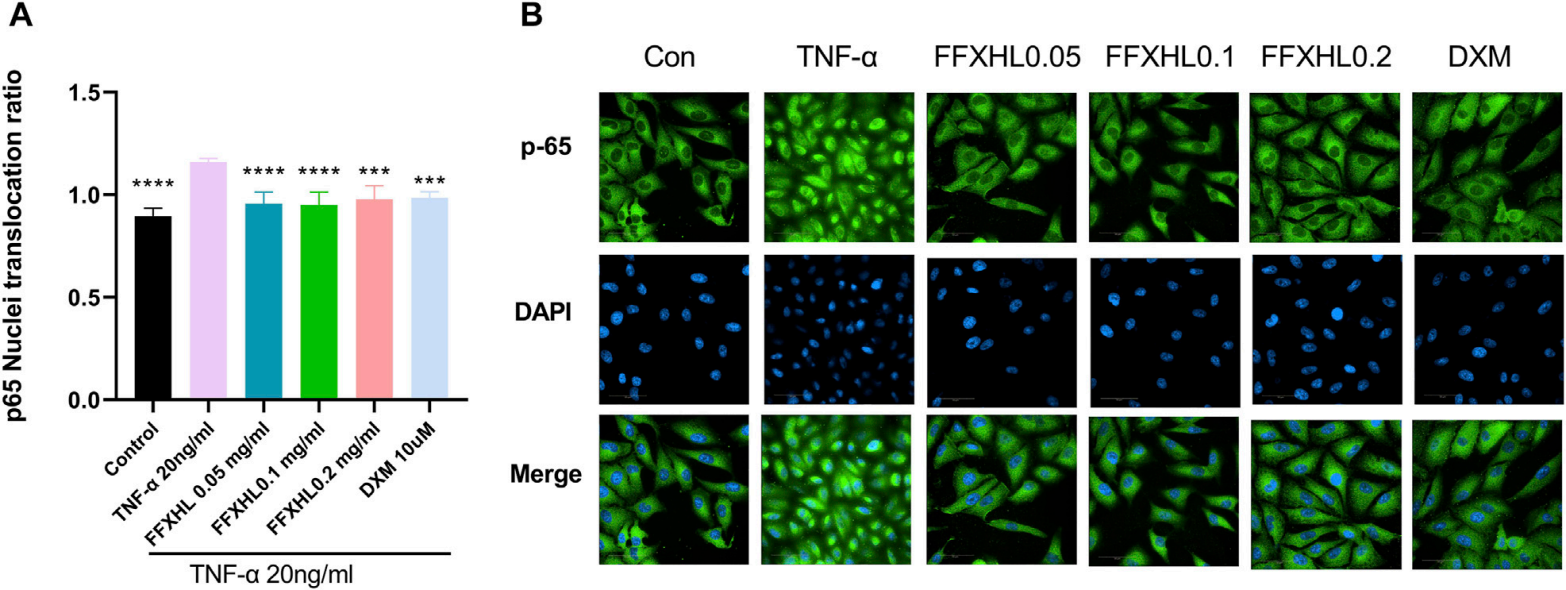
FFXHL inhibited the translocation of NF-κB p65. MH7A cells were pre-treated with different concentrations of FFXHL for 24 h, then the cells were stimulated with 20 ng/mL TNF-α. Indirect immunofluorescence with the specific anti-NF-κB p65 antibody was performed. Pictures and data were analysed by Operetta CLS High Content Screening. Scale bar 50 μm. The data are expressed as means ± SEM, ***p < 0.001, ****p < 0.0001 vs. TNF-α 20 ng/mL.

To further investigate the translocation events that govern NF-κB p65 function by FFXHL, the immunofluorescence technique was used. The cells were treated with different concentrations of FFXHL before TNF-α induction and then incubated with NF-κB p65 antibody. As shown in Figures 8A,B, TNF-α challenge induced NF-κB p65 nuclear translocation, and the process of NF-κB from the cytoplasm into the nucleus was significantly suppressed in MH7A cells by FFXHL.

### 3.10 FFXHL inhibits TNF-α stimulated production of MMP in MH7A cells

**FIGURE 9:**
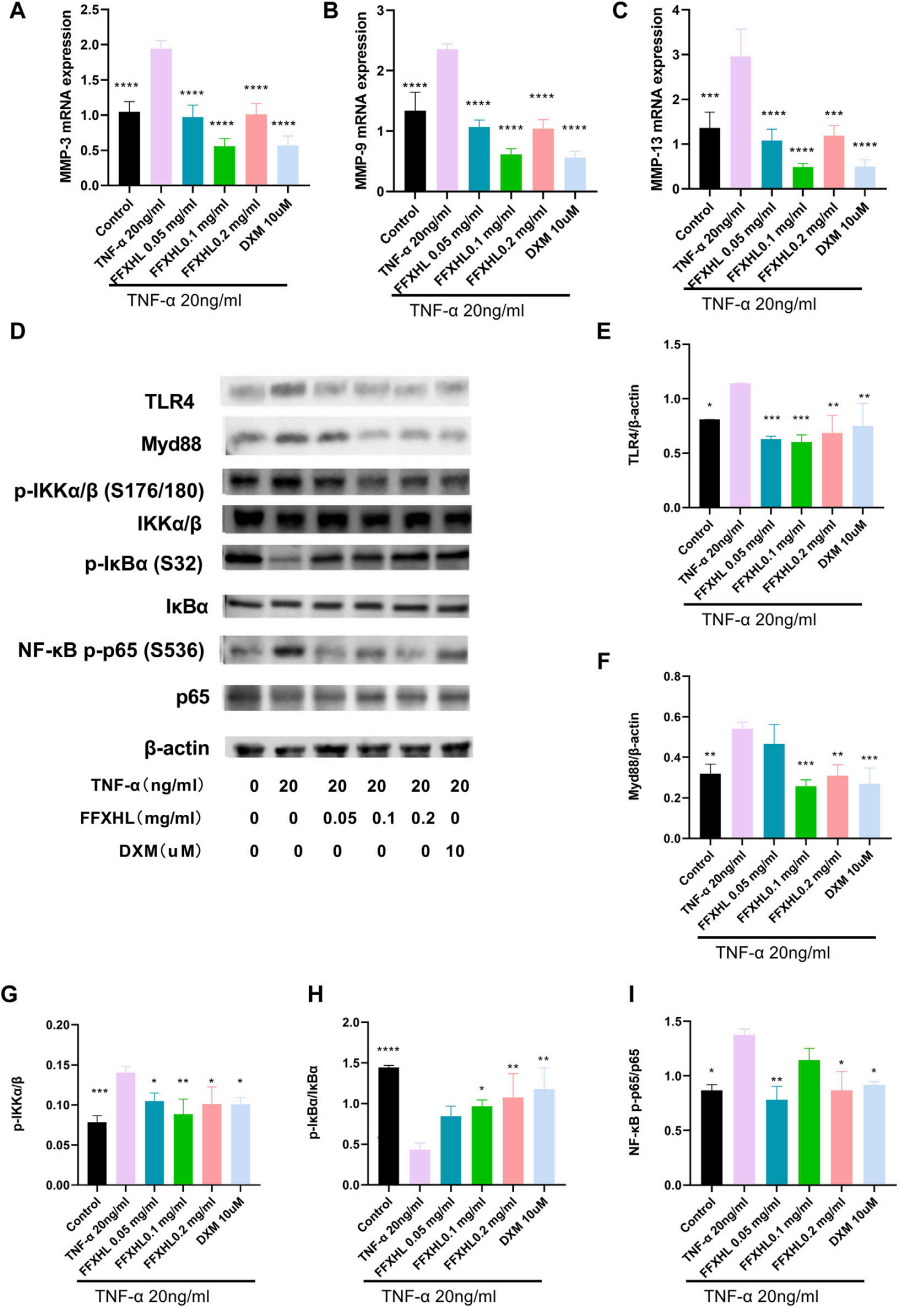
FFXHL inhibited the mRNA expression of MMP-3 **(A)**, MMP-9 **(B)** and MMP-13 **(C)** in TNF-α stimulated MH7A cells. Western blot analysis of different proteins in MH7A cells pre-treated with different concentrations of FFXHL for 24 h, then the cells were stimulated with 20 ng/mL TNF-α for 24 h. **(D)** Grey values of the TLR4, Myd88, p-IKKα/β, IKKα/β, p-IκBα, IκBα, NF-κB p-p65, p65 bands. **(E–I)** A graphic representation of protein expression of TLR4/Myd88/NF-κB pathway. The data are expressed as means ± SEM, *p < 0.05, **p < 0.01, ***p < 0.005, ****p < 0.0001 vs. TNF-α 20 ng/mL.

MMPs play a crucial role in the pathogenesis of RA by mediating joint cartilage and bone destruction. In this study, the mRNA levels of MMP-3, MMP-9, and MMP-13 were examined. After stimulation with 20 ng/mL TNF-α, the mRNA levels of MMP-3, MMP-9, and MMP-13 (Figures 9A–C) significantly increased in MH7A cells compared with the control group. However, in groups pretreated with FFXHL, the mRNA levels of these MMPs were significantly inhibited.

### 3.11 FFXHL inhibited theTLR4/MYD88/NF-κB, MAPKs and Akt pathways in MH7A cells

**FIGURE 10:**
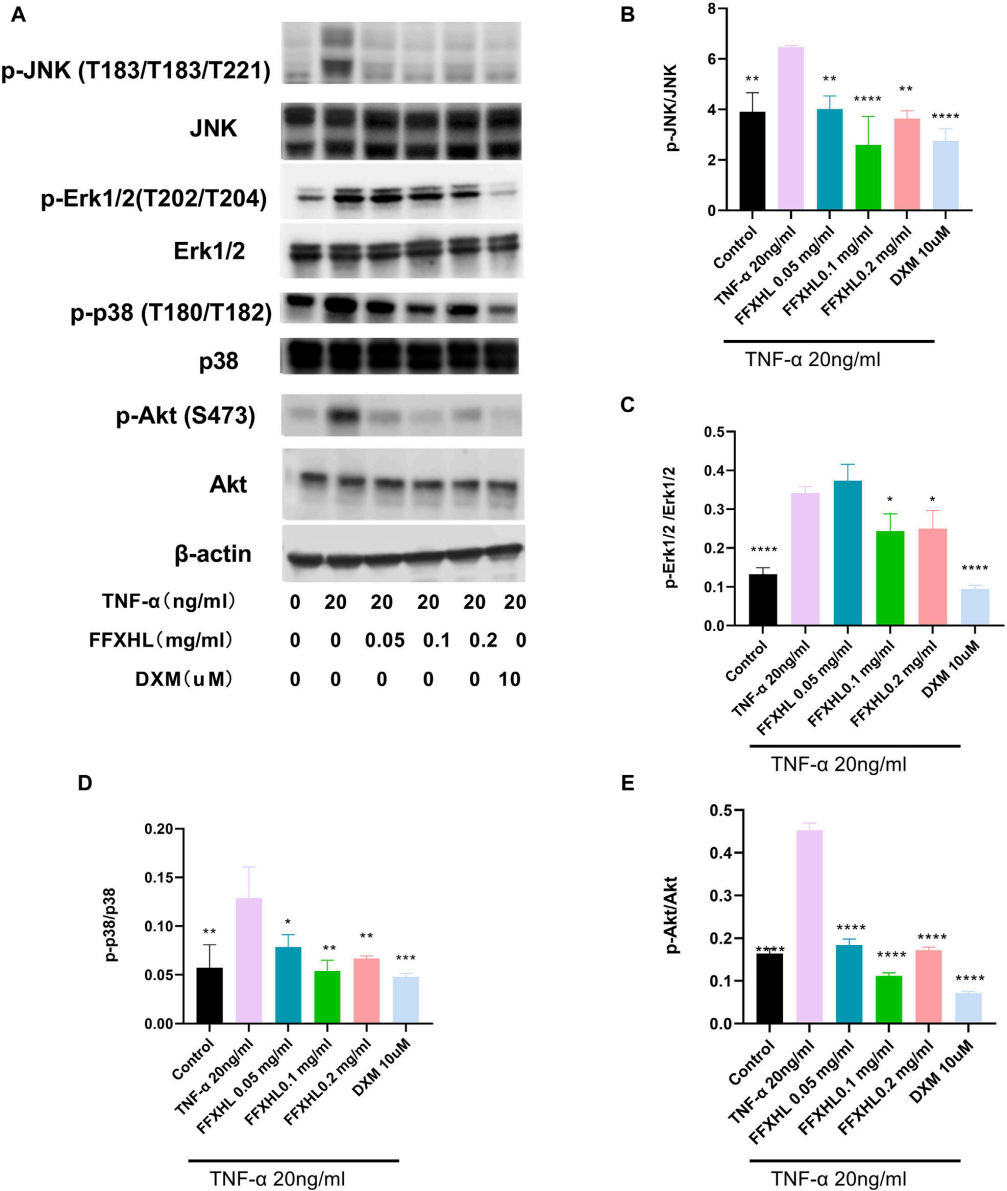
Western blot analysis of different proteins in MH7A cells pre-treated with different concentrations of FFXHL for 24 h, then the cells were stimulated with 20 ng/mL TNF-α for 24 h. **(A)** Gray values of the p-JNK, JNK, p-Erk1/2, Erk1/2, p-p38, p38, p-Akt, Akt. **(B–E)** A graphic representation of protein expression of JNK, Erk1/2, p38 and Akt. The data are expressed as means ± SEM, ***p < 0.005, ****p < 0.0001 vs. TNF-α 20 ng/mL.

To elucidate the mechanism of the anti-inflammatory effect of FHXHL in MH7A cells, the effects of FHXHL on inflammation-related signalling cascades, including TLR4/MyD88/NF-κB, Akt, and MAPKs pathways, were analysed. As shown in Figures 9D–I and Figures 10A–E, exposure of MH7A cells to TNF-α elevated the protein levels of p-Akt, p-NF-κB p65, TLR4, and MyD88, without affecting the total protein levels of Akt and NF-κB p65. Treatment with FFXHL attenuated this increase in MH7A cells (Figures 9E, F, I, 10E).

Furthermore, TNF-α significantly increased the phosphorylation levels of IKKα/β and the degradation of IκBα, but treatment with FFXHL strongly inhibited these phosphorylation and degradation events (Figures 9G, H). In Figures 10A–D, the results showed that after TNF-α stimulation, the MAPK pathway was activated, and the phosphorylation levels of p38, ERK, and JNK increased significantly, while treatment with FFXHL significantly inhibited their phosphorylation levels.

## 4 Discussion

RA is characterized by a systemic autoimmune pathology associated with a chronic inflammatory process (Radu and Bungau, 2021). TCM is commonly employed in China and other Asian countries for the treatment of rheumatic diseases, including RA. Approximately 75% of Chinese or Chinese immigrants prefer TCM or other complementary and alternative treatments for conditions like arthritis (Pan et al., 2019). While the anti-inflammatory and anti-rheumatic effects of certain Chinese medicine formulas or botanical drugs have been established (Song et al., 2020; Lu et al., 2023), many TCM formulations lack comprehensive pharmacological and mechanistic studies due to the distinct discovery processes of TCM and Western medicine. In this research, we aim to elucidate the therapeutic effect of FFXHL on RA using *in vivo* and *in vitro* experiments. The results demonstrate that FFXHL effectively alleviates arthritic symptoms in CIA rats and inhibits TNF-α stimulated inflammatory responses in MH7A cells. This could be attributed to FFXHL’s inhibition of TLR4/MYD88/NF-κB, MAPK and Akt pathways.

The CIA model shares clinical and histological characteristics with human RA, making it a common animal model for rheumatism research (Mcnamee et al., 2015). In our study, rats in the CIA model group exhibited notable redness and swelling of the hind paw, along with histological abnormalities in the synovial tissue, such as inflammatory cell infiltration and cartilage destruction. These findings align with other CIA model experiments (Zhai et al., 2018; Bai et al., 2020), confirming the successful establishment of the CIA model in our experiment. Following 20 days of FFXHL treatment, paw edema significantly decreased, and histological results supported FFXHL’s ability to inhibit the progression of CIA pathology by reducing inflammatory cell infiltration and cartilage injury. Additionally, FFXHL significantly increased the latency time of rats on the hot plate, indicating its pain-relieving effect. Numerous studies have emphasized the crucial role of inflammatory factors in RA pathology (Li et al., 2014; Zhai et al., 2018). Therefore, the reduction of inflammatory factor levels and regulation of the immune response represent effective strategies for RA treatment. In this study, FFXHL exhibited a therapeutic effect on RA, accompanied by a reduction in TNF-α, IL-1β and IL-6 production *in vivo*. Moreover, FFXHL elevated serum levels of the anti-inflammatory cytokine IL-10 in CIA rats. IL-10 is primarily produced by Th2 cells and can inhibit the production of pro-inflammatory cytokines. IL-10 has a protective role in RA, exerting anti-inflammatory effects in the CIA model (Min et al., 2004). These results collectively suggest that FFXHL demonstrates significant efficacy in both anti-inflammation and pain relief for RA treatment.

In the context of RA research, various well-established inflammatory pathways, including NF-κB, Akt, and MAPK, have been extensively studied. Given the lack of prior investigations into the mechanism of FFXHL, we selected these key pathways to illustrate how FFXHL alleviates RA. NF-κB influences the expression of numerous pro-inflammatory genes to regulate inflammation, synovial hyperplasia, and matrix degeneration (Zhai et al., 2017). Our results demonstrate that FFXHL robustly suppresses NF-κB protein levels *in vivo*, along with the phosphorylation and nuclear translocation of p65 in TNF-α stimulated MH7A cells. Additionally, FFXHL significantly inhibits TNF-α stimulated phosphorylation of IKKα/β and degradation of IκBα, which are crucial components of the NF-κB pathway. The TLR4/MyD88/NF-κB pathway has been implicated in the persistent inflammatory state characteristic of RA, contributing to joint tissue, cartilage, and bone destruction (Kong et al., 2020). In this study, FFXHL markedly decreased TNF-α stimulated protein levels of TLR4 and MyD88 in MH7A cells. As highlighted in the introduction, the MAPK pathway, activated by the TLR4-MyD88 complex, contributes to synovial inflammation by promoting the production of additional pro-inflammatory cytokines (Zhang et al., 2022). Given its pivotal role in RA inflammatory processes, the MAPK pathway has been a therapeutic target. FFXHL significantly reduced the phosphorylation of ERK1/2, JNK, and p38 in TNF-α stimulated MH7A cells, indicating that FFXHL’s partial anti-inflammatory effects may stem from its inhibition of the MAPK pathway. Moreover, the Akt pathway is involved in modulating immune responses and the inflammatory cascade in RA (Kong et al., 2020). Activated Akt has been demonstrated to phosphorylate and activate IKK, resulting in the degradation of IκB and the translocation of NF-κB to the nucleus (Bai et al., 2009). In the present study, the phosphorylation of Akt significantly increased in response to TNF-α, and FFXHL was observed to inhibit this phosphorylation in MH7A cells.

MMPs, zinc-dependent endopeptidases crucial for extracellular matrix reconstruction, play a role in RA pathogenesis by degrading joint non-collagen matrix components (Kim and Choi, 2010). FFXHL demonstrated the ability to inhibit TNF-α-induced expressions of MMP-3, MMP-9, and MMP-13 at the mRNA levels in MH7A cells. The regulation of MMP gene expression occurs through the MAPK signalling cascade in response to inflammatory signals binding to receptors in synovial cells or chondrocytes (Ni et al., 2019). FFXHL’s anti-inflammatory and MMP-inhibiting effects may be linked to its downregulation of ERK1/2, JNK, and p38 phosphorylation. ROS contribute to RA progression by activating MMPs and various inflammatory cytokines, and ROS in turn activate various inflammatory factors (Wang et al., 2020). TNF-α promotes intracellular ROS production, exacerbating inflammation-induced damage. FFXHL pre-treatment reversed TNF-α stimulated elevated ROS levels in MH7A cells, indicating a negative correlation with inflammation induced ROS generation and effective inhibition of oxidative damage.

TCM formulations, unlike conventional synthetic drugs, encompass multiple botanical drugs, enabling them to target various pathways with fewer side effects (Lü et al., 2015). Consequently, there is a growing interest in developing TCM drugs for RA. A typical TCM formulation consists of several medicinal botanical drugs or minerals, each with a specific role. In FFXHL, Aconiti radix (Chuan Wu, AC) and aconiti kusnezoffii radix (Cao Wu, AK) serve as “Jun” (Emperor) botanical drugs, playing a central role in the formula. Arisaema cum bile (Dan Nan Xing, ACB) functions as a “Chen (Minister)” botanical drugs. The function of Chen botanical drugs is to assist Jun botanical drugs in the treatment of the main pattern. “Zuo (Assistant)” botanical drugs, including myrrh (Mo Yao, CM) and olibanum (Ru Xiang, BC), reinforce the therapeutic effects of Jun and Chen botanical drugs while moderating or reducing their toxicity. Lastly, “Shi (Servant)” botanical drugs such as angelicae sinensis (Dang Gui, AS), chuanxiong rhizome (Chuan Xiong, LC), pheretima (Di Long), Paeoniae radix alba (Bai Shao, PA), and cyperi rhizoma (Xiang Fu, CR) guide the various botanical drugs to particular region of the body and harmonize the actions of the other botanical drugs.

As the primary botanical drugs of FFXHL, AC and AK contain aconitine, benzoylaconine, and aconine, exhibiting anti-inflammatory (Lee et al., 2022; Yu et al., 2020), analgesic (Wang et al., 2012), and immunomodulatory effects (Deng et al., 2018). Benzoylaconine, found in AC, has been shown to suppress IL-1β-induced expression of IL-6 and IL-8 by inhibiting the activation of MAPKs, Akt, and NF-κB pathways, suggesting its potential as a therapeutic agent for synovial inflammation in RA (Yu et al., 2020). ACB, a processed product with a history of over 900 years, has demonstrated anti-inflammatory effects by inhibiting pro-inflammatory cytokine production (Ahn and Je, 2012). BC and CM not only had anti-inflammatory effect, but also have analgesic effect. *Boswellia serrata* extract (BSE) has shown anti-inflammatory and analgesic properties (Sharma et al., 2010; Umar et al., 2014), and 85% ethanol extract of *Commiphora myrrha* demonstrated analgesic and anti-inflammatory activities (Su et al., 2011). AS (Wei et al., 2016), LC (Lyu and Shu, 2018), PA (Kong et al., 2018), and CR (Lu et al., 2022) have proven anti-inflammatory and analgesic effects. While research on FFXHL is limited, studies on its individual metabolites may support the formulation’s potential in RA treatment.

Currently used clinical drugs for RA, such as dexamethasone and methotrexate, may have side effects (Pan et al., 2019). Dexamethasone, as shown in this study, leads to a significant reduction in weight and spleen and thymus coefficients (Supplementary Figure S2). Methotrexate can cause liver problems, cirrhosis, and bone marrow deterioration (Bullock et al., 2019). In TCM formulations, different botanical drugs or processing methods are employed to mitigate side effects. The aconitine toxicity in AC and AK is reduced through steaming or boiling with hot water (Li et al., 2022). During FFXHL production, AC and AKR are soaked for 10–12 days and then boiled with liquorice and honeysuckle flower for 3–4 h, a traditional method to reduce aconitine toxicity (Liu et al., 2017; Li et al., 2019). Additionally, the combined use of PA with AC effectively reduces toxicity (Fan et al., 2012). The diverse botanical drugs in TCM formulations not only synergistically maximize efficacy but also counteract potential side effects.

## 5 Conclusion

**FIGURE 11:**
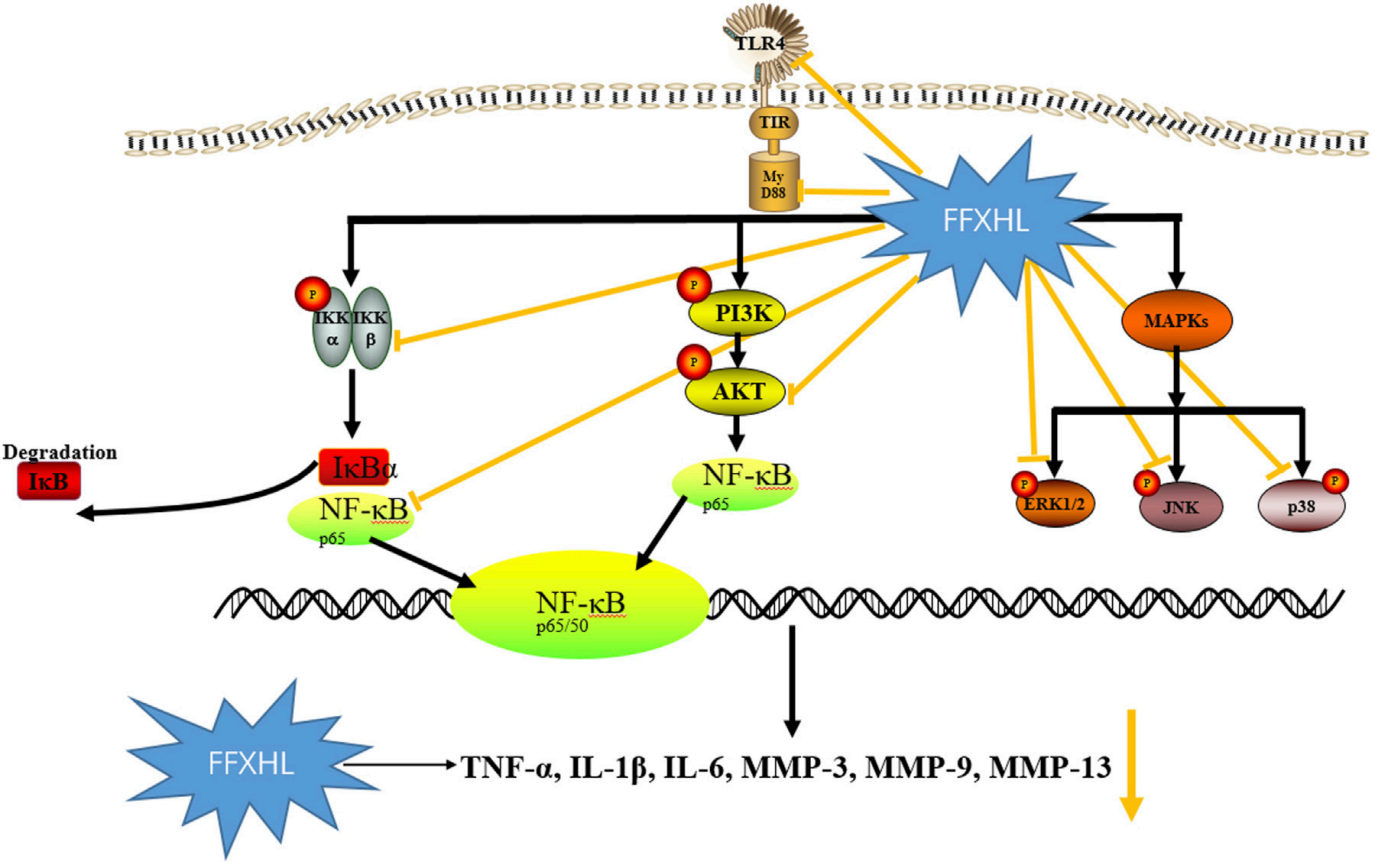
The schematic diagram illustrating the potential pathway associated with FFXHL inhibition of TNF-α stimulated inflammation. FFXHL suppresses TNF-α stimulated pro-inflammatory cytokines production through 1) inhibition the protein level of TLR4 and Myd88, inhibition the phosphorylation of p-Akt, p-IKKα/β and the degradation of IκBα, which may suppress the NF-κB p65 translocate into the nucleus. 2) inhibition the phosphorylation of p42, p38 and JNK MAPK, this may contribute to reduce the MMP-3, MMP-9, and MMP-13 gene expressions. These results suggest that the therapeutic effects of FFXHL on RA may be associated with the regulation of the TLR4/MYD88/NF-κB, MAPK, and Akt pathways.

In summary, this study marks the first demonstration of the treatment effect of FFXHL pills on RA both *in vivo* and *in vitro*. The findings reveal that FFXHL significantly attenuate joint swelling, diminishes the production of systemic pro-inflammatory factors, and reduces NF-κB p65 levels in CIA rats. Moreover, FFXHL inhibits the production of pro-inflammatory factors and MMPs, as well as the translocation of NF-κB p65, in TNF-α induced MH7A cells. Mechanistic insights suggest that the therapeutic effects of FFXHL on RA may be associated with the regulation of the TLR4/MYD88/NF-κB, MAPK, and Akt pathways (Figure 11).

## Data Availability

The original contributions presented in the study are included in the article/Supplementary Material, further inquiries can be directed to the corresponding author.
