# Supplementary material for: Anti-rheumatoid arthritis effects of traditional Chinese medicine Fufang Xiaohuoluo pill on collagen-induced arthritis rats and MH7A cells
Source: Front Pharmacol. 2024 Apr 29;15:1374485. doi: 10.3389/fphar.2024.1374485 (PMC11089244; doi:10.3389/fphar.2024.1374485)
Supplement: Supplementary file 1 [file DataSheet1.docx]

Table S1 All components in FFXHL pill

| **Botanical plant names** | **Family** | **Drug names** | **Part used and weight used (commercial use)** |
| --- | --- | --- | --- |
| *Aconiturn carmichaelii* Debeaux | Ranunculaceae | Aconiti Radix | Dried parent root tuber |
|  |  |  | 750 gram |
| *Aconitum kusnezoffii* Reichb. | Ranunculaceae | Aconiti Kusnezoffii Radix | Dried root tuber |
|  |  |  | 750 gram |
| *Arisaema erubescens*（Wall.）Schott | Araceae | Arisaema Cum Bile | Arisaema Cum Bile is produced by fermenting a mixture of powdered Arisaema erubescens（Wall.）Schott with bile from cows, sheep, or pigs |
|  |  |  | 750 gram |
| *Angelica sinensis* (Oliv.) Diels | Apiaceae | Angelicae Sinensis | Radix dried root |
|  |  |  | 500 gram |
| *Ligusticum chuanxiong* Hort | Apiaceae | chuanxiong rhizoma | Dried rhizome |
|  |  |  | 500 gram |
| *Pheretima aspergillum* (E.Perrier) | Megascolecidae | Pheretima | 375 gram |
| *Paeonia lactiflora* Pall. | Paeoniaceae | Paeoniae Radix Alba | Dried root |
|  |  |  | 250 gram |
| *Boswellia carterii* Birdw. | Burseraceae | Olibanum | Resin |
|  |  |  | 375 gram |
| *Commiphora myrrha* Engl. | Burseraceae | myrrh | Dried resin |
|  |  |  | 375 gram |
| *Cyperus rotundus* L. | Cyperaceae | cyperi rhizoma | Dried rhizome |
|  |  |  | 500 gram |

Table S2 All components in Xiaohuoluo and FFXHL pills

| **Xiaohuoluo pill** | **FFXHL pill** |
| --- | --- |
| Radix Aconiti (Chuan Wu) | Aconiti Radix (Chuan Wu) |
| Aconiti Kusnezoffii Radix (Cao Wu) | Aconiti Kusnezoffii Radix (Cao Wu) |
| Arisaema Cum Bile (Dan Nan Xing) | Arisaema Cum Bile (Dan Nan Xing) |
| Pheretima (Di Long) | Pheretima (Di Long) |
| Olibanum (Ru Xiang) | Olibanum (Ru Xiang) |
| Myrrh (Mo Yao) | Myrrh (Mo Yao) |
|  | Paeoniae Radix Alba (Bai Shao) |
|  | Chuanxiong rhizome (Chuan Xiong) |
|  | Cyperi rhizoma (Xiang Fu) |
|  | Angelicae Sinensis (Dang Gui) |


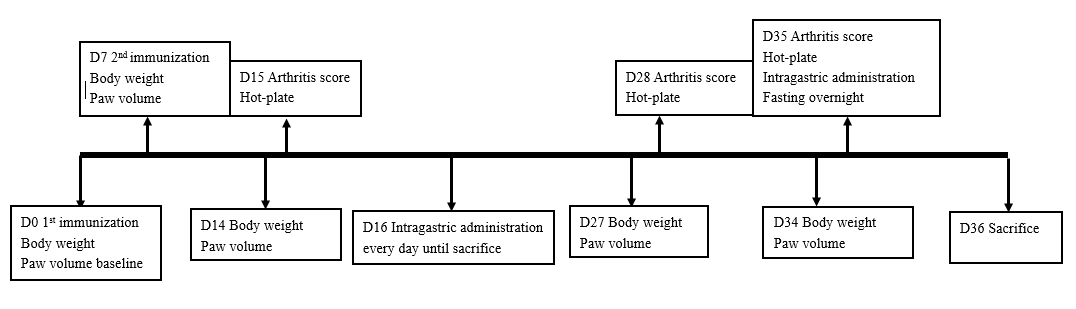


**Figure S1.** Experimental schedules of the development of the collagen-induced arthritis mouse model and drug treatment.

Table S3 Retention time, multiple reaction monitoring (MRM) transitions and parameters for the determination of four compounds

| **Name** | **RT(min)** | **Transition**  **monitored**  **Q1 (m/z)** | **Transition**  **monitored**  **Q3 (m/z)** | **Declustering potential (V)** | **Collision**  **energy(V)** |
| --- | --- | --- | --- | --- | --- |
| Benzoylhypacoitine | 6.63 | 574.1 | 542.3 | 160 | 48 |
| Benzoylmesaconine | 6.21 | 590.3 | 540.4 | 170 | 50 |
| Benzoylaconitine | 6.46 | 604.3 | 554.5 | 170 | 53 |
| Paeoniflorin | 3.81 | 479.1 | 121.1 | -120 | -25 |

Table S4 Sequences of primers used in real-time PCR

| **Gene** |  | **Primer sequences (5′-3′)** |
| --- | --- | --- |
| MMP3 | F | CCATCTCTTCCTTCAGGCGT |
|  | R | ATGCCTCTTGGGTATCCAGC |
| MMP9 | F | CGCAGACATCGTCATCCAGT |
|  | R | GAAATGGGCGTCTCCCTGAA |
| IL-1β | F | AACCTCTTCGAGGCACAAGG |
|  | R | GGCGAGCTCAGGTACTTCTG |
| IL-6 | F | CCACCGGGAACGAAAGAGAA |
|  | R | GAGAAGGCAACTGGACCGAA |
| MMP13 | F | ATGCAGCAAGCTCCATGACT |
|  | R | ATCAGGAACCCCGCATCTTG |
| Actin | F | GTCATTCCAAATATGAGATGCGT |
|  | R | GCTATCACCTCCCCTGTGTG |

Table S5 Antibodies employed for Western blotting analyses

| **Name** | **Species** | **Dilutions** |
| --- | --- | --- |
| TLR4 | Rabbit | 1:500 |
| Myd88 | Rabbit | 1:500 |
| p65 | Rabbit | 1:500 |
| p-p65 | Rabbit | 1:500 |
| p-IKKα/β | Rabbit | 1:300 |
| IKKα/β | Rabbit | 1:300 |
| p-JNK1/2/3 | Rabbit | 1:300 |
| JNK1/2/3 | Rabbit | 1:300 |
| p-Erk1/2 | Mouse | 1:1000 |
| Erk1/2 | Rabbit | 1:1000 |
| p-p38 | Rabbit | 1:1000 |
| p38 | Rabbit | 1:1000 |
| β-actin | Rabbit/ Mouse | 1:1000 |
| p-IκBα | Rabbit | 1:300 |
| IκBα | Rabbit | 1:300 |
| p-Akt | Mouse | 1:1000 |
| Akt | Rabbit | 1:1000 |

Table S6 The information of identified compound

| **Component name** | **Formula** | **Neutral mass (Da)** | **Observed m/z** | **Ms error ppm** | **Observed RT (min)** | **Response** | **Mode** |
| --- | --- | --- | --- | --- | --- | --- | --- |
| Benzoylmesaconine | C31H43NO10 | 589.2887 | 590.2957 | -0.4 | 21 | 13620697 | +H |
| 3β-Hydroxy-11-oxo-olean-12-en-28-oic acid | C30H46O4 | 470.33961 | 471.3465 | -0.8 | 51.29 | 9881071 | -H +H |
| Cholic acid | C24H40O5 | 408.28757 | 407.2809 | 1.4 | 33.48 | 8408717 | -H |
| Oleanolic acid | C30H48O3 | 456.36035 | 455.3536 | 1.2 | 63.74 | 7319009 | -H +H |
| 14-Benzoylaconine | C32H45NO10 | 603.30435 | 604.3116 | 0 | 22.31 | 7184398 | +H |
| Glycodeoxycholic acid | C26H43NO5 | 449.31412 | 448.3075 | 1.5 | 29.91 | 6684240 | -H |
| Benzoylhypaconine | C31H43NO9 | 573.29378 | 574.3007 | -0.5 | 23.24 | 5995552 | +H |
| Hederagenin | C30H48O4 | 472.35526 | 471.3479 | -0.2 | 50.97 | 5308903 | -H |
| Glycocholic acid | C26H43NO6 | 465.30904 | 464.3023 | 1.1 | 28.18 | 4784549 | -H |
| Mesaconitine | C24H39NO6 | 437.27774 | 438.2848 | -0.5 | 14.15 | 4395409 | +H |
| Hypaconitine | C33H45NO10 | 615.30435 | 616.3113 | -0.4 | 27.92 | 3744169 | +H |
| Mesaconitine | C33H45NO11 | 631.29926 | 632.3069 | 0.6 | 26.05 | 3651318 | +H |
| α-Boswellic acid | C30H48O3 | 456.36035 | 455.3534 | 0.8 | 62.95 | 3556209 | -H +H |
| β-Boswellic acid | C30H48O3 | 456.36035 | 455.3525 | -1.2 | 59.52 | 3199966 | -H +H |
| Neoline | C24H39NO6 | 437.27774 | 438.2851 | 0.2 | 13.79 | 2938170 | +H |
| α-Cyperone | C15H22O | 218.16707 | 219.1741 | -1.3 | 44.53 | 2881086 | +H |
| Betulinic acid | C30H48O3 | 456.36035 | 455.3534 | 0.8 | 68.47 | 2731155 | -H |
| Acetyl-α-boswellic acid | C32H50O4 | 498.37091 | 497.3635 | -0.3 | 65.87 | 2635668 | -H +H |
| Rel-1S,2S-epoxy-4R-furanogermacr-10(15)-en-6-one | C15H18O3 | 246.12559 | 247.1329 | 0.2 | 28.32 | 2572434 | +H |
| Rifampicin | C43H58N4O12 | 822.40512 | 821.3973 | -0.6 | 30.89 | 2145936 | -H |
| 23-Hydroxy-betulinic acid | C30H48O4 | 472.35526 | 471.3477 | -0.6 | 55.04 | 2061694 | -H |
| Aconitine | C34H47NO11 | 645.31491 | 646.3218 | -0.6 | 27.87 | 1930691 | +H |
| 15-α-Hydroxyneoline | C24H39NO7 | 453.27265 | 454.2799 | 0 | 13.56 | 1802951 | +H |
| E-Ligustilide | C12H14O2 | 190.09938 | 191.1068 | 0.7 | 49.14 | 1727329 | +H |
| Curculigoside G | C22H26O10 | 450.1526 | 449.1454 | 0.3 | 15.72 | 1664555 | -H |
| Talatisamine | C24H39NO5 | 421.28282 | 422.29 | -0.2 | 15.51 | 1657073 | +H |
| 4-Hydroxy-3-butylphthalide | C12H14O3 | 206.09429 | 207.1017 | 0.8 | 20.79 | 1406576 | +H |
| 3β-Hydroxy-11α,12α-epoxyolean-28-13β-olide | C30H46O5 | 486.33452 | 485.328 | 1.6 | 53.72 | 1307171 | -H +H |
| (−)-Calamenene | C15H22 | 202.17215 | 203.1793 | -0.5 | 44.15 | 1222171 | +H |
| Verticiol | C20H34O | 290.26097 | 291.268 | -0.7 | 60.03 | 1120415 | +H |
| Linolenic acid | C18H30O2 | 278.22458 | 279.2316 | -0.9 | 44.04 | 1102583 | +H |
| Beiwutine | C33H45NO12 | 647.29418 | 648.3018 | 0.5 | 24.25 | 1079114 | +H |
| Acetyl-β-boswellic acid | C32H50O4 | 498.37091 | 497.3637 | 0.2 | 66.84 | 1057565 | -H +H |
| Arachidonic acid | C20H32O2 | 304.24023 | 305.2472 | -1 | 39.37 | 1033643 | +H |
| Albiflorin | C23H28O11 | 480.16316 | 479.1561 | 0.4 | 15.72 | 955829 | -H |
| Z-Ligustilide | C12H14O2 | 190.09938 | 191.1067 | 0 | 48.23 | 938938 | +H |
| Deoxyaconitine | C34H47NO10 | 629.32 | 630.3274 | 0.2 | 29.79 | 893558 | +H |
| Acsonine | C31H41NO8 | 555.28322 | 556.2911 | 1.1 | 26.17 | 696074 | +H |
| Citric acid | C6H8O7 | 192.027 | 191.021 | 6.6 | 2.66 | 671911 | -H |
| β-Cyperone | C15H22O | 218.16707 | 219.1742 | -0.6 | 42.04 | 667388 | +H |
| Rel-3R-methoxy-4S-furanogermacra-1E,10(15)-dien-6-one | C16H20O3 | 260.14124 | 261.1477 | -3 | 43.43 | 636093 | +H |
| Paeoniflorin | C23H28O11 | 480.16316 | 479.1563 | 0.8 | 20.38 | 628205 | -H |
| Chasmanine | C25H41NO6 | 451.29339 | 452.3 | -1.4 | 16.86 | 583486 | +H |
| 11α,12α-Epoxy-23β,23-dihydroxyolean-28,13β-olide | C30H46O6 | 502.32944 | 501.3231 | 1.8 | 47.92 | 563575 | -H |
| Paeoniflorigenone | C17H18O6 | 318.11034 | 319.1179 | 0.8 | 14.96 | 512248 | +H |
| Senkyunolide | C12H16O2 | 192.11503 | 193.1225 | 1 | 34.78 | 452261 | +H |
| α-Rotunol | C15H22O2 | 234.16198 | 235.1691 | -0.9 | 28.38 | 438726 | +H |
| 9-Octadecenoic acid | C18H34O2 | 282.25588 | 281.2494 | 2.7 | 60.86 | 431126 | -H |
| Chlorogenic acid | C16H18O9 | 354.09508 | 353.0888 | 2.7 | 12.14 | 419234 | -H |
| Paeonilactone B | C10H12O4 | 196.07356 | 197.081 | 0.9 | 14.97 | 416763 | +H |
| Licuraside | C26H30O13 | 550.16864 | 549.1616 | 0.4 | 17.62 | 414985 | -H |
| 3,5-Dicaffeoylquinic acid | C25H24O12 | 516.12678 | 515.1202 | 1.3 | 19.11 | 404810 | -H |
| Neoligustilide | C12H14O2 | 190.09938 | 191.1069 | 1.4 | 35.58 | 397341 | +H |
| Piscidic acid | C11H12O7 | 256.0583 | 255.0521 | 4.1 | 10.82 | 396688 | -H |
| (+)-Sebiferine | C20H23NO4 | 341.16271 | 340.1559 | 1.2 | 14.26 | 342572 | -H |
| Liquiritigenin | C15H12O4 | 256.07356 | 257.081 | 0.7 | 17.67 | 321170 | -H +H |
| D-Mannose | C6H12O6 | 180.06339 | 179.057 | 4.9 | 3.19 | 294654 | -H |
| Nootkatone | C15H22O | 218.16707 | 219.1742 | -0.5 | 36.86 | 291266 | +H |
| Gluconic acid | C6H12O7 | 196.0583 | 195.0521 | 5.4 | 3.19 | 287517 | -H |
| Foresticine | C24H39NO6 | 437.27774 | 438.2844 | -1.4 | 15.86 | 281440 | +H |
| β-Rotunol | C15H22O2 | 234.16198 | 235.169 | -1.2 | 41.67 | 258124 | +H |
| Dehydroabietic acid | C20H28O2 | 300.20893 | 301.2159 | -0.9 | 50.27 | 255522 | +H |
| 2-Furoic acid | C5H4O3 | 112.01604 | 111.0093 | 4.8 | 2.66 | 242513 | -H |
| Sandaracopimaric acid | C20H30O2 | 302.22458 | 303.2312 | -2.3 | 41.16 | 238656 | +H |
| Nonanedioic acid | C9H16O4 | 188.10486 | 187.0986 | 5.6 | 20.1 | 224758 | -H |
| 5-Hydroxymethyl-2-furaldehyde | C6H6O3 | 126.03169 | 127.0386 | -2.5 | 1.21 | 221535 | +H |
| Liquiritin | C21H22O9 | 418.12638 | 417.1192 | 0.1 | 17.73 | 215261 | -H |
| Patchoulenone | C15H22O | 218.16707 | 219.1741 | -1.3 | 43.93 | 214132 | +H |
| Palmitic acid | C16H32O2 | 256.24023 | 255.2334 | 1.6 | 59.76 | 211656 | -H |
| Ferulic acid | C10H10O4 | 194.05791 | 193.0513 | 3.6 | 17.5 | 197135 | -H |
| 3β-Hydroxyolean-12-en-28-al | C30H48O2 | 440.36543 | 441.3703 | -5.4 | 64.95 | 193365 | +H |
| 3-Phenylpropionic acid | C9H11NO2 | 165.07898 | 164.0724 | 4.3 | 7.05 | 189897 | -H |
| Paeonilactone C | C17H18O6 | 318.11034 | 319.1178 | 0.4 | 20.39 | 187215 | +H |
| (-)-α-Selinene | C15H24 | 204.1878 | 205.1947 | -1.8 | 43.81 | 175637 | +H |
| 30-Norhederagenin | C29H44O4 | 456.32396 | 455.3166 | -0.2 | 49.48 | 174243 | -H |
| Aconifine | C34H47NO12 | 661.30983 | 662.3174 | 0.5 | 26.4 | 174038 | +H |
| Gallic acid | C7H6O5 | 170.02152 | 169.0151 | 5 | 3.12 | 160780 | -H |
| Bergapten | C12H8O4 | 216.04226 | 215.0337 | -6.1 | 1.11 | 158428 | -H |
| abietic acid | C20H30O2 | 302.22458 | 303.2311 | -2.6 | 31.48 | 156329 | +H |
| Guaiacol | C5H5N5 | 135.0545 | 136.0617 | -0.2 | 1.65 | 151954 | +H |
| Epilupeol | C30H50O | 426.38617 | 427.3905 | -7 | 64.2 | 150025 | +H |
| Arginine | C6H14N4O2 | 174.11168 | 175.1189 | -0.2 | 1.06 | 141014 | +H |
| Aristolone | C15H22O | 218.16707 | 219.1739 | -2 | 39.41 | 129361 | +H |
| Corymboside | C26H28O14 | 564.14791 | 563.1417 | 1.9 | 15.61 | 120422 | -H |
| Apigenin-6-C-galac-tosyl-8-C-arabinoside | C26H28O14 | 564.14791 | 563.1417 | 1.9 | 15.61 | 120422 | -H |
| 3-Methoxy-4-hydroxybenzoic acid | C8H8O4 | 168.04226 | 167.0357 | 4.4 | 13.22 | 115942 | -H |
| 6-Hydroxypurine | C5H4N4O | 136.03851 | 137.046 | 1.7 | 3.36 | 109175 | +H |
| (3R-trans)-4-Ethenyl-4-methyl-3-(1-methylethenyl)-1-(methylethyl)-cyclohexene | C15H24 | 204.1878 | 205.1948 | -1.5 | 48.36 | 99596 | +H |
| Benzoylpaeoniflorin | C30H32O12 | 584.18938 | 583.1851 | 5.2 | 25.95 | 97125 | -H |
| Eudesma-4(14),11-diene | C15H24 | 204.1878 | 205.1946 | -2.1 | 47.82 | 92620 | +H |
| Rotundene | C15H24 | 204.1878 | 205.1947 | -1.7 | 45.69 | 82194 | +H |
| Oxypaeoniflorin | C23H28O12 | 496.15808 | 495.1516 | 1.7 | 12.11 | 81989 | -H |
| Sebacic acid | C10H18O4 | 202.12051 | 201.1145 | 6.4 | 23.47 | 81261 | -H |
| Senkyunone | C22H30O2 | 326.22458 | 327.2307 | -3.5 | 58.91 | 73644 | +H |
| Lycoctonine | C25H41NO7 | 467.2883 | 468.2947 | -1.9 | 14.05 | 72517 | +H |
| Tyrosine | C9H11NO3 | 181.07389 | 182.0815 | 1.8 | 1.52 | 70154 | +H |
| Linolic acid | C18H32O2 | 280.24023 | 279.2336 | 2.3 | 47.53 | 67030 | -H |
| Mesaconitine | C33H45NO11 | 631.29926 | 632.306 | -0.8 | 22.79 | 66472 | +H |
| α-Copaene (Copaene) | C15H24 | 204.1878 | 205.1943 | -3.9 | 45.26 | 66326 | +H |
| Spathulenol | C15H24O | 220.18272 | 221.1898 | -0.8 | 29.16 | 66144 | +H |
| Eudesma-4(14),11-dien-3β-ol | C15H24O | 220.18272 | 221.1898 | -0.8 | 29.16 | 66144 | +H |
| Neocnidilide | C12H18O2 | 194.13068 | 195.137 | -4.9 | 21.23 | 65910 | +H |
| Vanillin | C8H8O3 | 152.04734 | 153.0547 | 0.4 | 18.65 | 64548 | +H |
| 2-Methoxy-5-acetoxy-fruranogermacr-1(10)-en-6-one | C18H24O5 | 320.16237 | 321.1696 | -0.3 | 39.51 | 63459 | +H |
| Catenarin | C15H10O6 | 286.04774 | 285.0408 | 1 | 24.07 | 63087 | -H +H |
| Z-6,8′,7,3′-Diligustilide | C24H28O4 | 380.19876 | 381.2055 | -1.5 | 43 | 61317 | +H |
| α-Cubebene | C15H24 | 204.1878 | 205.1945 | -3 | 61.2 | 59257 | +H |
| Isoliquiritigenin | C15H12O4 | 256.07356 | 255.0671 | 3.3 | 29.38 | 57692 | -H +H |
| Butylated hydroxytoluene | C15H24O | 220.18272 | 221.1896 | -1.8 | 53.58 | 57604 | +H |
| Rel-2R-methyl-5S-acetoxy-4R-furano-germacr-1(10)Z-en-6-one | C18H24O5 | 320.16237 | 321.1697 | 0.1 | 32.39 | 57104 | +H |
| 1-O-β-D-Glucopyranosylpaeonisuffrone | C16H24O9 | 360.14203 | 359.1342 | -1.5 | 3.43 | 56399 | -H |
| Isoschaftoside | C26H28O14 | 564.14791 | 563.1418 | 2.2 | 16.09 | 55742 | -H |
| Guaiacol | C7H8O2 | 124.05243 | 123.0456 | 3.9 | 13.22 | 55192 | -H |
| Olibanumols I | C29H48O2 | 428.36543 | 429.3706 | -4.9 | 69.14 | 54195 | +H |
| Brefeldin A | C16H24O4 | 280.16746 | 281.1731 | -6 | 46.23 | 53478 | +H |
| Paeonol | C9H10O3 | 166.06299 | 165.0562 | 2.8 | 16.19 | 52540 | -H |
| Schaftoside | C26H28O14 | 564.14791 | 565.1557 | 0.9 | 15.6 | 52485 | -H +H |
| Guanine | C5H5N5O | 151.04941 | 152.0571 | 2.8 | 3.35 | 50843 | +H |
| Phthalic anhydride | C8H4O3 | 148.01604 | 149.0233 | 0 | 0.72 | 50583 | +H |
| furfural | C5H4O2 | 96.02113 | 97.0281 | -3.3 | 1.16 | 49377 | +H |
| N-CARBOBENZOXY-DL-LEUCINE | C6H13NO2 | 131.09463 | 132.1015 | -3.4 | 2.13 | 49070 | +H |
| Protocatechuic acid | C7H6O4 | 154.02661 | 153.0199 | 3.9 | 8.27 | 47267 | -H |
| (Z)-(1S,5R)-β-Pinen-10-yl-β-vicianoside | C21H34O10 | 446.2152 | 445.2079 | 0 | 22.87 | 46912 | -H |
| Palbinone | C22H30O4 | 358.21441 | 357.208 | 2.5 | 30.77 | 46557 | -H |
| Lactinolide | C10H16O4 | 200.10486 | 199.0984 | 4 | 22.36 | 46214 | -H |
| Valine | C5H11NO2 | 117.07898 | 118.0858 | -3.7 | 1.49 | 45976 | +H |
| β-Copaene | C15H24 | 204.1878 | 205.1947 | -1.8 | 47.14 | 45535 | +H |
| Cyperene | C15H24 | 204.1878 | 205.1947 | -1.8 | 47.14 | 45535 | +H |
| 3,4-Dihydroxycinnamic acid | C9H8O4 | 180.04226 | 179.0363 | 7.2 | 13.27 | 44765 | -H |
| 1-Galloyl-β-D-glucose | C13H16O10 | 332.07435 | 331.0683 | 3.6 | 7 | 44441 | -H |
| succinic acid | C4H6O4 | 118.02661 | 117.0196 | 2.1 | 1.79 | 44086 | -H |
| Cnidilide | C12H18O2 | 194.13068 | 195.137 | -4.7 | 22.92 | 43817 | +H |
| Xiongterpene | C39H54O5 | 602.39712 | 601.3908 | 1.6 | 61.68 | 42572 | -H |
| β-Elemene | C15H24 | 204.1878 | 205.1945 | -2.7 | 59.54 | 41804 | +H |
| 11α,12α-Epoxy-3β,23-dihydroxy-30-norolean-20(29)-en-28,13β-olide | C29H42O5 | 470.30322 | 469.2946 | -2.8 | 35.3 | 41181 | -H |
| Apigenin-6,8-di-C-β-D-galactoside | C27H30O15 | 594.15847 | 593.1505 | -1.2 | 15.73 | 39479 | -H |
| Isocyperol | C15H24O | 220.18272 | 221.1898 | -0.9 | 49.95 | 38123 | +H |
| Rotundone | C15H24O | 220.18272 | 221.1898 | -0.9 | 49.95 | 38123 | +H |
| 1,8-Dihydroxy-3-methylanthraquinone (Chrysophanol) | C15H10O4 | 254.05791 | 253.0512 | 2.4 | 22.84 | 36531 | -H +H |
| Thymol | C10H14O | 150.10447 | 151.1115 | -1.4 | 53.57 | 35935 | +H |
| Cyperol | C15H24O | 220.18272 | 221.1898 | -0.8 | 24.84 | 35192 | +H |
| 6-O-β-D-Glucopyranosyllactinolide | C16H26O9 | 362.15768 | 361.1497 | -1.9 | 9.65 | 34965 | -H |
| Perilla alcohol | C10H16O | 152.12012 | 153.1273 | -0.8 | 53.57 | 34441 | +H |
| Vicenin Ⅱ | C27H30O15 | 594.15847 | 593.1507 | -0.9 | 15.42 | 32370 | -H |
| D-Alanine | C3H7NO2 | 89.04768 | 90.0547 | -3.3 | 1.16 | 31647 | +H |
| Benzoic acid | C7H6O2 | 122.03678 | 121.03 | 3.8 | 12.34 | 31540 | -H |
| benzyl alcohol | C7H8O | 108.05751 | 109.0648 | 0.1 | 42.11 | 29696 | +H |
| Alloocimene | C10H16 | 136.1252 | 137.1324 | -0.5 | 44.04 | 29196 | +H |
| Kaempferol-3,7-di-O-β-D-glucoside | C27H30O16 | 610.15338 | 609.1474 | 2.1 | 17.63 | 28571 | -H |
| L-aspartic acid | C4H7NO4 | 133.03751 | 132.0309 | 5.1 | 1.13 | 27669 | -H |
| Nicotinic acid | C6H5NO2 | 123.03203 | 124.0389 | -3 | 1.46 | 24724 | +H |
| Carvacrol | C10H14O | 150.10447 | 151.1115 | -1.4 | 46.02 | 23746 | +H |
| Rel-2R-methoxy-4R-furanogermacr-1(10)E-en-6-one | C16H22O3 | 262.15689 | 263.1638 | -1.2 | 39.36 | 22701 | +H |
| Stigmasterol | C29H48O | 412.37052 | 413.3751 | -6.5 | 62.49 | 21746 | +H |
| Kaempferol-3-O-β-D-glucopyranoside | C21H20O11 | 448.10056 | 447.0932 | -0.3 | 18.25 | 19898 | -H |
| Ethyl salicylate | C9H10O3 | 166.06299 | 165.0563 | 3.4 | 11.15 | 19079 | -H |
| Apocynin | C9H10O3 | 166.06299 | 165.0564 | 3.9 | 15.42 | 16312 | -H |
| Oxybenzoyl paeoniflorin | C30H32O13 | 600.18429 | 599.1782 | 1.9 | 22.17 | 15183 | -H |
| Sugetriol triacetate | C21H30O6 | 378.20424 | 377.1996 | 6.9 | 31.21 | 13966 | -H |
| Paeonilactone A | C10H14O4 | 198.08921 | 197.0822 | 1.5 | 15.61 | 12905 | -H |
| Lactiflorin | C23H26O10 | 462.1526 | 461.1456 | 0.7 | 1.27 | 10839 | -H |


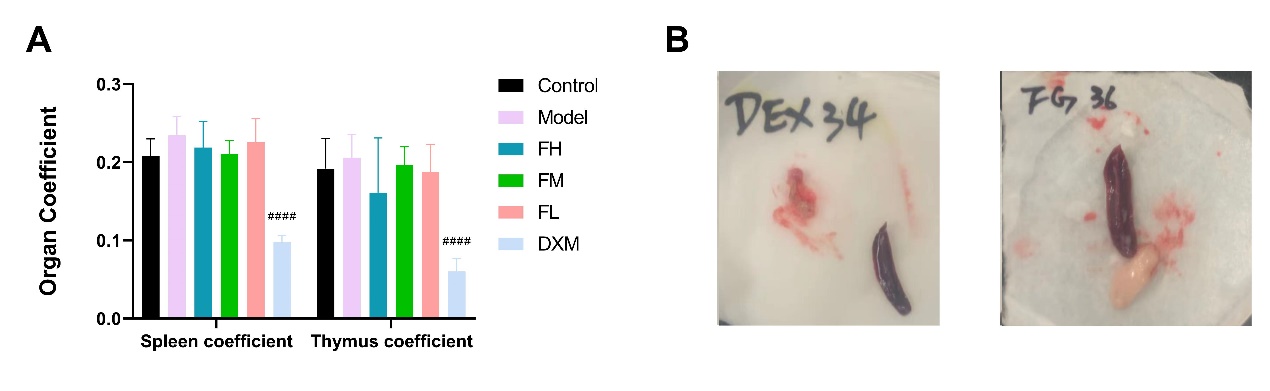


Figure S2. Effect of FFXHL and DXM on spleen and thymus coefficient of CIA rats. #### P < 0.0001 vs control group; DEX=DXM, FG=FH.
